# VTA glutamatergic projections to the nucleus accumbens suppress psychostimulant-seeking behavior

**DOI:** 10.1038/s41386-024-01905-3

**Published:** 2024-06-26

**Authors:** M. Flavia Barbano, Jia Qi, Emma Chen, Uzma Mohammad, Orlando Espinoza, Marcos Candido, Huiling Wang, Bing Liu, Suyun Hahn, François Vautier, Marisela Morales

**Affiliations:** Integrative Neuroscience Research Branch, National Institute on Drug Abuse, National Institutes of Health, Baltimore, MD 21224 USA

**Keywords:** Addiction, Motivation

## Abstract

Converging evidence indicates that both dopamine and glutamate neurotransmission within the nucleus accumbens (NAc) play a role in psychostimulant self-administration and relapse in rodent models. Increased NAc dopamine release from ventral tegmental area (VTA) inputs is critical to psychostimulant self-administration and NAc glutamate release from prelimbic prefrontal cortex (PFC) inputs synapsing on medium spiny neurons (MSNs) is critical to reinstatement of psychostimulant-seeking after extinction. The regulation of the activity of MSNs by VTA dopamine inputs has been extensively studied, and recent findings have demonstrated that VTA glutamate neurons target the NAc medial shell. Here, we determined whether the mesoaccumbal glutamatergic pathway plays a role in psychostimulant conditioned place preference and self-administration in mice. We used optogenetics to induce NAc release of glutamate from VTA inputs during the acquisition, expression, and reinstatement phases of cocaine- or methamphetamine-induced conditioned place preference (CPP), and during priming-induced reinstatement of cocaine-seeking behavior. We found that NAc medial shell release of glutamate resulting from the activation of VTA glutamatergic fibers did not affect the acquisition of cocaine-induced CPP, but it blocked the expression, stress- and priming-induced reinstatement of cocaine- and methamphetamine CPP, as well as it blocked the priming-induced reinstatement of cocaine-seeking behavior after extinction. These findings indicate that in contrast to the well-recognized mesoaccumbal dopamine system that is critical to psychostimulant reward and relapse, there is a parallel mesoaccumbal glutamatergic system that suppresses reward and psychostimulant-seeking behavior.

## Introduction

The mesocorticolimbic dopamine system plays a critical role in psychostimulant conditioned place preference (CPP), self-administration, and reinstatement of psychostimulant-seeking after extinction [1–4]. This system is integrated by dopamine neurons located in the ventral tegmental area (VTA) and their efferents to cortex and limbic structures, such as the nucleus accumbens (NAc) [5, 6]. Pioneering studies established that addictive drugs increase NAc release of dopamine [7, 8] which modulates activity of local medium spiny neurons (MSNs); further studies showed that the activity of MSNs is also regulated by glutamatergic inputs, critical to reinstatement of drug-seeking after extinction [3, 9, 10]. Indeed, drug-seeking responses to cocaine are highly regulated by NAc glutamatergic inputs from the prefrontal cortex (PFC) [11–13], and the ventral hippocampus [14], both synapsing on MSNs.

We previously demonstrated that in addition to the well-known mesocorticolimbic dopamine system, there is a parallel mesocorticolimbic glutamatergic system derived from VTA glutamatergic neurons that preferentially innervate the NAc shell [15, 16]. In contrast to cortical and hippocampal glutamatergic inputs synapsing on MSNs, VTA glutamatergic neurons establish excitatory synapses with a subset of parvalbumin (PV) GABAergic interneurons distributed in the NAc medial shell (mShell) [16]. Although PV interneurons represent a small fraction of the NAc neuronal population [17], they exert a strong regulation of MSNs by establishing multiple synapses on their soma and dendrites [18]. Feedforward inhibition by PV interneurons regulates MSNs’ spike timing, and the high activation of these interneurons results in MSNs’ decreased firing [19]. Given that glutamatergic activation of MSNs reinstates cocaine-seeking behavior and the fact that VTA-glutamatergic neurons provide a strong glutamatergic input to a subset of NAc PV-GABAergic interneurons, which drives the inhibition of MSNs, this raises the possibility that VTA glutamatergic inputs play a role in cocaine-seeking behavior by inducing the release of GABA within the NAc mShell.

Here, we determined the extent to which VTA glutamatergic inputs to NAc mShell (mesoaccumbal glutamatergic system) play a role in cocaine-seeking behavior. We found that NAc photoactivation of axons from VTA glutamatergic neurons did not affect the acquisition of cocaine-induced CPP but inhibited the expression and reinstatement of both cocaine-and methamphetamine-induced CPP. We further confirmed the inhibitory effects of NAc release of glutamate from VTA glutamatergic inputs on the reinstatement of cocaine-seeking induced by drug priming injections in a cocaine self-administration study. Given that the NAc receives inputs from some VTA neurons that co-release dopamine and glutamate [15, 20], we examined whether VTA projections to the NAc mShell releasing dopamine alone or co-releasing glutamate and dopamine played a role in the expression or reinstatement of cocaine-induced CPP and found this not to be the case. We conclude that VTA neighboring dopamine and glutamate neurons innervating the NAc mShell play different roles in psychostimulant-seeking behavior. These results, together with the previous demonstration that glutamatergic neurons are present in the human VTA [21], may open new avenues to further investigate whether the mesoaccumbal glutamatergic pathway is a suitable target to decrease relapse rates in people who use psychostimulants.

## Materials and methods

A complete description of the materials and methods used can be found as Supplementary information.

### Subjects

Male and female mice were bred in the NIDA/IRP animal facility (Baltimore, MD) and were used in behavioral and anatomical experiments. Animal care and use were in strict accordance with institutional and international standards and were approved by the National Institute on Drug Abuse Animal Care and Use Committee (ASP: 21-INRB-2).

### Surgeries

A detailed description of the surgeries performed can be found as Supplementary information.

### RNAscope in situ hybridization combined with immunolabeling and histological verification

A detailed description of in situ hybridization and immunohistological procedures conducted can be found as Supplementary information.

### Drugs

Cocaine hydrochloride and methamphetamine hydrochloride were obtained from the National Institute on Drug Abuse (NIDA) Drug Supply Program through the NIDA Intramural Research Program pharmacy and were dissolved in sterile 0.9% physiological saline.

### Apparatus

#### Conditioned place preference

A three-chamber place conditioning apparatus was used for the behavioral experiments (ANY-box, Stoelting, Wood Dale, IL). The position of the animal was monitored via an overhead closed-circuit camera interfaced with video tracking software (ANY-maze, Stoelting).

#### Operant behavior

Drug self-administration training and testing occurred in operant chambers (Model ENV-307W-C, MED Associates, Inc., St. Albans, VT) equipped with two retractable levers. One of them was selected as the reinforced lever for delivering the drug and the other was selected as the non-reinforced lever. Active pressing on the reinforced lever resulted in a cocaine infusion while pressing on the non-reinforced lever did not result in cocaine infusion. A stimulus light, located above the reinforced lever, and a 75 dB tone (2000 Hz) were paired contingently with the delivery of the drug.

### Behavioral studies

A detailed description of the behavioral studies conducted can be found as Supplementary information.

### Electrophysiology

A detailed description of the electrophysiological recordings conducted can be found as Supplementary Information.

### Quantification and statistical analysis

Results are presented as mean ± SEM, with behavioral data analyzed using a multifactorial analysis of variance (MANOVA) with group (eYFP or ChR2-eYFP) as the between-subjects factor, and days, trials, or phases of testing as within-subject factors. When the same mice were tested under different conditions, a repeated measures ANOVA was used instead. Post-hoc analyses were performed using the Newman–Keuls test when the initial *p* value was significant. A result was considered significant if *p* < 0.05. All data were analyzed using Statistica software (Statsoft Inc., Tulsa, OK).

## Results

### NAc release of glutamate from VTA-VGluT2 fibers does not affect acquisition of cocaine CPP but inhibits the expression and reinstatement of cocaine CPP

**Fig. 1:**
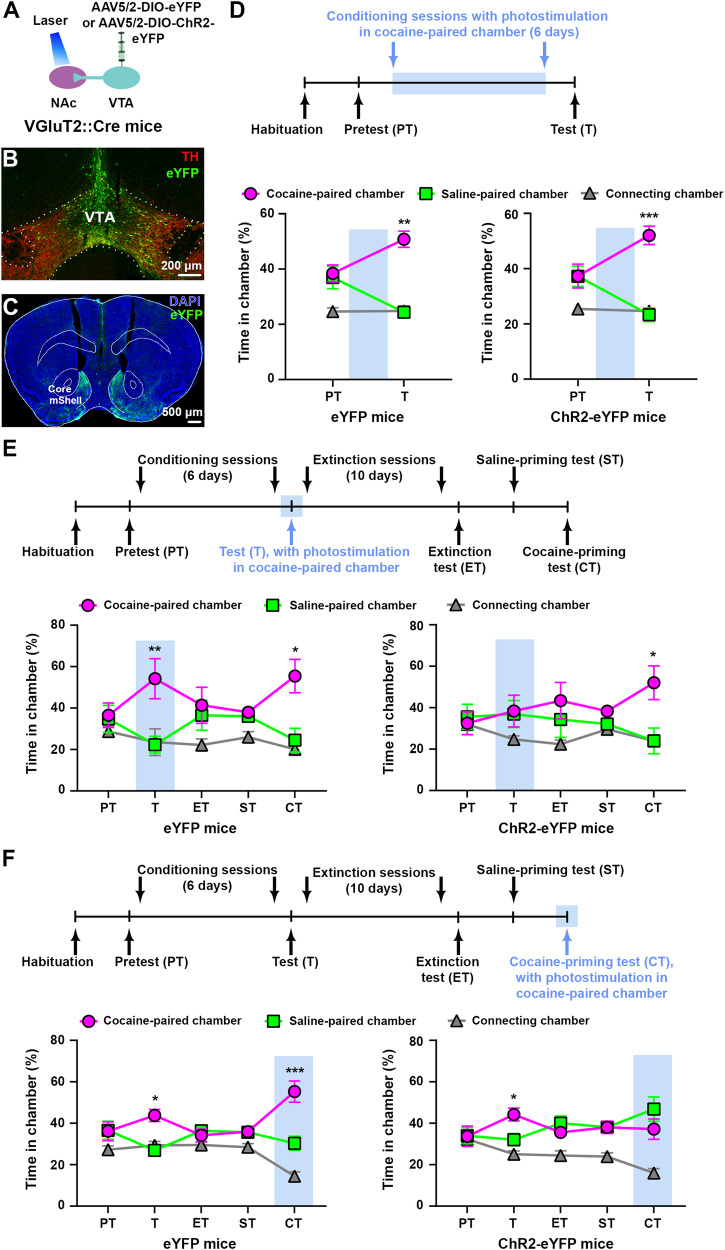
NAc shell release of glutamate from VTA-VGluT2 fibers inhibits the expression and reinstatement of cocaine CPP. **A** VTA injection of AAV5/2-DIO-eYFP or AAV5/2-DIO-ChR2-eYFP and NAc shell optic fibers. **B** VTA immunofluorescence detection of eYFP-expressing neurons (green) and TH (red). **C** eYFP fibers from VTA-VGluT2 neurons (green) in the NAc and optic fiber placements. **D** Cocaine CPP acquisition timeline (top). Both eYFP (*n* = 6) and ChR2-eYFP mice (*n* = 8) spent more time in the cocaine-paired chamber after the conditioning sessions (bottom; eYFP: chamber × experimental phase: F_2,10_ = 6.05, *p* < 0.05; ChR2-eYFP: F_2,14_ = 12.62, *p* < 0.001, ANOVA with Newman–Keuls post hoc-test). **E** Cocaine CPP expression timeline (top). ChR2-eYFP mice (*n* = 6) spent less time in the cocaine-paired chamber than eYFP control mice (*n* = 6) during photostimulation test (T). Both groups showed reinstatement of cocaine CPP during the cocaine-priming test (CT, bottom; eYFP: chamber × experimental phase: F_8,40_ = 3.36, *p* < 0.01; ChR2_-_eYFP: F_8,40_ = 3.51, *p* < 0.01, ANOVA with Newman–Keuls post-hoc test). **F** Cocaine CPP reinstatement timeline (top). ChR2-eYFP mice (*n* = 9) spent less time in the cocaine-paired chamber than eYFP control mice (*n* = 8) during the cocaine-priming test (CT) paired with photostimulation (bottom; eYFP: chamber × experimental phase: F_8,56_ = 6.92, *p* < 0.001; ChR2-eYFP: F_8,64_ = 3.57, *p* < 0.01, ANOVA with Newman–Keuls post-hoc test_)_. *p < 0.05, **p < 0.01, ***p < 0.001, against saline-paired chamber. Light-blue rectangles indicate photostimulation. ChR2 channelrhodopsin-2, Core nucleus accumbens core, DAPI 4’,6-diamidino-2-phenylindol, eYFP enhanced yellow fluorescent protein, mShell nucleus accumbens medial shell, TH tyrosine hydroxylase, VTA ventral tegmental area.

We targeted VTA-VGluT2 neurons and their axons by injecting a Cre-inducible adeno-associated virus (AAV) with a double-floxed inverted open reading frame (DIO) expressing channelrhodopsin-2 (ChR2; ChR2-eYFP mice) tethered to an enhanced yellow fluorescent protein (eYFP) in the VTA of VGluT2::Cre mice (Fig. 1A, B, Supplementary Fig. 1). Control mice were injected with a vector lacking ChR2 (eYFP mice), and optic fibers were bilaterally implanted dorsal to the NAc of ChR2-eYFP and eYFP mice (Fig. 1C, Supplementary Fig. 2). By fluorescent microscopy, we found that VTA fibers expressing eYFP were localized predominantly in the medial shell (mShell) of the NAc (Fig. 1C, Supplementary Fig. 2B–E), consistent with our previous studies demonstrating that VTA-VGluT2 neurons mostly innervate the NAc mShell and activate glutamatergic receptors on PV-interneurons [16]. To determine the role of midbrain VTA-NAc glutamatergic pathway activation in the acquisition of cocaine-induced CPP, we tested these mice in a CPP procedure using a three-chamber apparatus in which they received continuous trains of NAc photostimulation at 20 Hz during the conditioning sessions with cocaine (15 mg/kg; Fig. 1D). We found that both ChR2-eYFP and eYFP control mice spent more time in the cocaine-paired chamber after the conditioning sessions (Fig. 1D, Supplementary Fig. 3A–C) and did not detect aversion in ChR2-eYFP mice. Next, to confirm that NAc release of glutamate from VTA inputs induced place aversion prior to cocaine exposure, we prepared new cohorts of ChR2-eYFP and eYFP mice and tested their behavior in a three-chamber apparatus in which one chamber was paired with photostimulation. While mice had access to all 3 chambers, they received continuous trains of NAc photostimulation (20 Hz) each time they entered the laser-paired chamber. As we previously reported [16], eYFP control mice spent a similar amount of time in each chamber but ChR2-eYFP mice avoided the laser-paired chamber (Supplementary Figs. 2F, 4A–C). We then tested the same cohort of mice by applying the cocaine-induced CPP acquisition procedure and found that both ChR2-eYFP and eYFP mice spent more time in the cocaine-paired chamber, although this cocaine-paired chamber was associated with photostimulation during the conditioning sessions (Supplementary Fig. 4D, E). These findings indicate that NAc release of glutamate from VTA-VGluT2 fibers induces aversion but does not play a role in the acquisition of cocaine-induced CPP.

In other cohorts of mice, we determined the extent to which NAc glutamate release from VTA-VGluT2 fibers plays a role in the expression of cocaine-induced CPP. After completion of the conditioning sessions, mice received continuous trains of photostimulation (20 Hz) whenever they entered (and for as long as they remained) in the cocaine-paired chamber (Fig. 1E). We observed that eYFP mice, but not ChR2-eYFP mice, displayed a significant preference for the cocaine-paired chamber (Fig. 1E, Supplementary Fig. 3D, E), suggesting that NAc release of glutamate from VTA-VGluT2 fibers participates in the expression of cocaine-induced CPP. Next, we determined the extent to which the observed inhibitory effect was due to a deficit in expression of the preference for the cocaine-paired chamber or a disruption in the consolidation of memory for the cocaine-chamber association. We trained both ChR2-eYFP and eYFP mice in extinction conditions for 10 days, followed by an extinction test, a saline-priming test, and a subsequent cocaine-priming test (15 mg/kg; Fig. 1E). We observed a lack of preference for either chamber by both ChR2-eYFP and eYFP mice on the extinction and saline-priming test days. In contrast, both ChR2-eYFP and eYFP mice spent significantly more time in the cocaine-paired chamber after a cocaine-priming injection (Fig. 1E, Supplementary Fig. 3E). Collectively these findings indicate that while NAc release of glutamate from VTA-VGluT2 fibers inhibits the expression of cocaine-induced CPP, it does not appear to affect memory for the association between cocaine and the chamber in which it was administered.

Next, we determined whether NAc release of glutamate from VTA-VGluT2 fibers plays a role in the reinstatement of cocaine CPP. For these studies, we used a cohort of mice in which the cocaine CPP procedure was done without laser stimulation and NAc photostimulation was restricted to the step in which mice accessed the cocaine-paired chamber during the cocaine-priming test (Fig. 1F). Both ChR2-eYFP and eYFP mice showed a preference for the cocaine-paired chamber after the conditioning sessions, a preference that was no longer observed after extinction training or during the saline-priming test. While a priming injection of cocaine (15 mg/kg) reinstated cocaine-induced CPP in eYFP mice, it did not induce reinstatement in ChR2-eYFP mice (Fig. 1F, Supplementary Fig. 3F–H). These findings indicate that NAc release of glutamate from VTA-VGluT2 fibers is sufficient to disrupt cocaine priming-induced reinstatement of cocaine CPP.

Given that the above-mentioned studies were conducted in male mice, we next determined the extent to which the inhibitory effect of NAc release of glutamate from VTA fibers on the expression and reinstatement of cocaine-seeking behavior also occurred in female mice. We trained ChR2-eYFP and eYFP female mice in the cocaine CPP procedure (Supplementary Figs. 2G and 5). The eYFP female mice showed a preference for the cocaine-paired chamber after the conditioning sessions when NAc photostimulation was contingent to visits to the chamber. In contrast, ChR2-eYFP female mice did not show preference for the cocaine-paired chamber during NAc photostimulation. While both groups of mice displayed reinstatement of cocaine CPP after a cocaine-priming injection in the absence of NAc photostimulation, only eYFP female mice showed reinstatement of cocaine CPP after a cocaine injection when NAc photostimulation was administered (Supplementary Fig. 5C). These results indicate that the inhibitory effect of NAc release of glutamate from VTA-VGluT2 fibers on the expression and reinstatement of cocaine CPP is not sex-dependent. Thus, we used both male and female mice in the remaining experiments.

In another cohort of mice, we determined the extent to which the inhibitory effect of NAc glutamate release from VTA-VGluT2 fibers on the expression and reinstatement of cocaine CPP was affected by the dose of cocaine used. We did not detect behavioral differences among mice injected with 5 or 10 mg/kg of cocaine; in both tested doses, NAc release of glutamate from VTA-VGluT2 fibers disrupted the expression and priming-induced reinstatement of cocaine CPP in ChR2-eYFP mice but not in eYFP mice (Supplementary Figs. 1H and 6A–D). In a different cohort of mice, we injected mice with saline (saline-priming injection) and tested them in the presence or absence of photostimulation. We confirmed that a saline-priming injection in the absence of NAc photostimulation did not induce any chamber preference in ChR2-eYFP or eYFP mice. In contrast, a saline-priming injection promoted the preference for the cocaine-paired chamber in ChR2-eYFP, but not in eYFP mice when NAc photostimulation was paired with the saline chamber (Supplementary Fig. 6E). Thus, it appears that the preference for the cocaine-paired chamber in ChR2-eYFP mice was, in part, a result of the aversion induced in the saline chamber by NAc photostimulation. In contrast to saline-priming effects, we found that reinstatement of cocaine CPP was more pronounced in ChR2-eYFP mice after a cocaine-priming injection in the absence of NAc photostimulation. We further confirmed that in the presence of NAc photostimulation, eYFP, but not ChR2-eYFP mice, showed reinstatement of cocaine CPP after a cocaine-priming injection (Supplementary Fig. 6E).

**Fig. 2:**
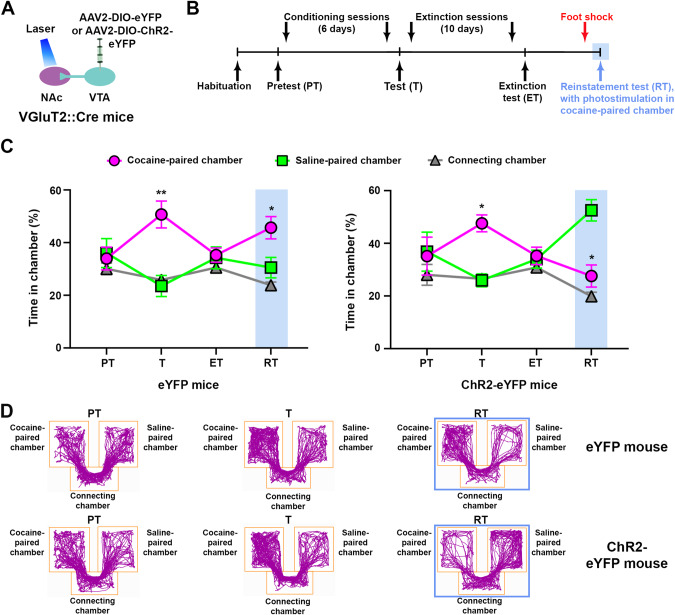
NAc release of glutamate from VTA-VGluT2 fibers inhibits stress-induced reinstatement of cocaine CPP. **A** VTA injection of AAV2-DIO-eYFP or AAV2-DIO-ChR2-eYFP and NAc shell optic fibers. **B** Stress-induced reinstatement timeline. **C** While eYFP control mice (*n* = 7) showed reinstatement of cocaine CPP during the stress-induced reinstatement test (RT) in the presence of NAc photostimulation, ChR2-eYFP mice (*n* = 7) avoided the cocaine-paired chamber during the stress-induced reinstatement test (RT) paired with NAc photostimulation (eYFP: chamber × experimental phase: F_6,36_ = 3.03, *p* < 0.05; ChR2-eYFP: chamber × experimental phase: F_6,36_ = 5.30, *p* < 0.001, ANOVA with Newman–Keuls post-hoc test). *p < 0.05, **p < 0.01, against saline-paired chamber. Light-blue rectangles indicate photostimulation. **D** Track plots from an eYFP (top) and a ChR2-eYFP (bottom) mouse during pretest (PT), expression test (T) and stress-induced reinstatement test (RT) in the presence of NAc photostimulation. Blue boxes indicate photostimulation.

To determine the extent to which the inhibitory effect of NAc glutamate release from VTA-VGluT2 fibers generalizes to other factors inducing reinstatement, we trained ChR2-eYFP and eYFP mice in the cocaine CPP procedure (Fig. 2) and after extinction, we ran a stress-induced reinstatement test. Mice received 15 foot-shocks over 15 min (1 shock/min with a variable inter-shock interval, 0.5 s duration, 0.8 mA) and then were immediately placed in the CPP apparatus, where NAc photostimulation was administered upon entrance to (and for as long as the mice remained in) the cocaine-paired chamber. While eYFP control mice reinstated cocaine-induced CPP after the foot-shock session, reinstatement was blocked in ChR2-eYFP mice (Fig. 2C, D). Collectively, these findings indicate that NAc release of glutamate from VTA-VGluT2 fibers plays a dose-independent inhibitory role in the expression and reinstatement of cocaine CPP in both male and female mice, but it does not play a role in the acquisition of cocaine-induced CPP.

### Glutamate from VTA-VGluT2-only neurons inhibits the expression and reinstatement of cocaine-induced CPP

All the above-described experiments were done by targeting the whole population of VTA-VGluT2 neurons in VGluT2::Cre mice. However, the VTA-VGluT2 neurons are heterogenous: some release solely glutamate (VGluT2-only), others co-release glutamate and dopamine (VGluT2-TH), or co-release glutamate and GABA [22]. While we had previously demonstrated that the dual VTA-glutamate-GABA neurons do not target the NAc, the dual VTA-VGluT2-TH neurons constitute near 20% of the population of VTA neurons projecting to the NAc mShell [16]. Thus, to determine the extent to which the NAc projections from VTA subpopulations of VGluT2-only or dual VGluT2-TH neurons contributed to inhibition in the expression and reinstatement of cocaine-induced CPP, we crossed VGluT2::Cre and TH::Flpo mice to generate double recombinase VGluT2::Cre-TH::Flpo mice. Then, we injected INTRSECT (intronic recombinase sites enabling combinatorial targeting) viral vectors into the VTA of these VGluT2::Cre-TH::Flpo mice to access specific VTA neuronal subpopulations (Supplementary Fig. 7A).

To drive the expression of eYFP in the VTA subpopulation of VGluT2-TH neurons, we injected AAV-C_ON_/F_ON_-ChR2-eYFP vectors into the VTA of VGluT2::Cre-TH::Flpo mice. Under this condition, the expression of eYFP requires the presence of Cre recombinase (henceforth referred to as C_ON_) and Flp recombinase (henceforth referred to as F_ON_) within the same neuronal population. In addition, we targeted VTA VGluT2-only neurons by intra-VTA injection of AAV-C_ON_/F_OFF_ vectors (requiring the presence of Cre recombinase and the absence of Flp recombinase), and by intra-VTA injections of AAV-C_OFF_/F_ON_ vectors (requiring the absence of Cre recombinase and the presence of Flp recombinase), we targeted VTA-TH-only neurons (Supplementary Fig. 7A). After confirming VTA neuronal expression of eYFP (Supplementary Fig. 7B, C), we examined the extent to which VGluT2 mRNA and TH mRNA colocalized in the total population of VTA eYFP neurons (Supplementary Fig. 7B, C). By combination of immunohistochemistry and RNAscope, we found that within the total population of VTA neurons in which we drove the expression of eYFP in TH-VGluT2 neurons (by VTA injection of the C_ON_/F_ON_-ChR2-eYFP vector), more than 83% expressed both VGluT2 and TH mRNAs (713/839 neurons; 4 mice; Supplementary Fig. 7H), ≈9% expressed only VGluT2 mRNA (72/839 neurons) and ≈6.5% expressed only TH mRNA (43/839 neurons). In mice receiving intra-VTA injections of the C_ON_/F_OFF_-ChR2-eYFP vector to drive the expression of eYFP in VGluT2-only neurons, we detected that around 61% of the VTA-eYFP neurons expressed only VGluT2 mRNA (566/982 neurons; 3 mice; Supplementary Fig. 7D, E, I), ≈2% expressed only TH mRNA (22/982 neurons), and ≈8% expressed both VGluT2 and TH mRNAs (76/982 neurons). In mice injected with the C_OFF_/F_ON_-ChR2-eYFP vector to drive the expression of eYFP in VTA-TH-only neurons, we found that within the total population of neurons expressing eYFP, around 75% expressed only TH mRNA (409/545 neurons; 3 mice; Supplementary Fig. 7F, G, J), ≈1.5% expressed only VGluT2 mRNA (8/545 neurons), and ≈2% expressed both VGluT2 and TH mRNAs (12/545 neurons). The high frequency of expression of VGluT2, TH, or co-expression of VGluT2 and TH mRNAs in eYFP-expressing neurons indicates that the use of INTRSECT viral vectors in combination with the newly developed VGluT2::Cre-TH::Flpo mouse line is a reliable strategy to target and study diverse VTA neuronal populations.

**Fig. 3:**
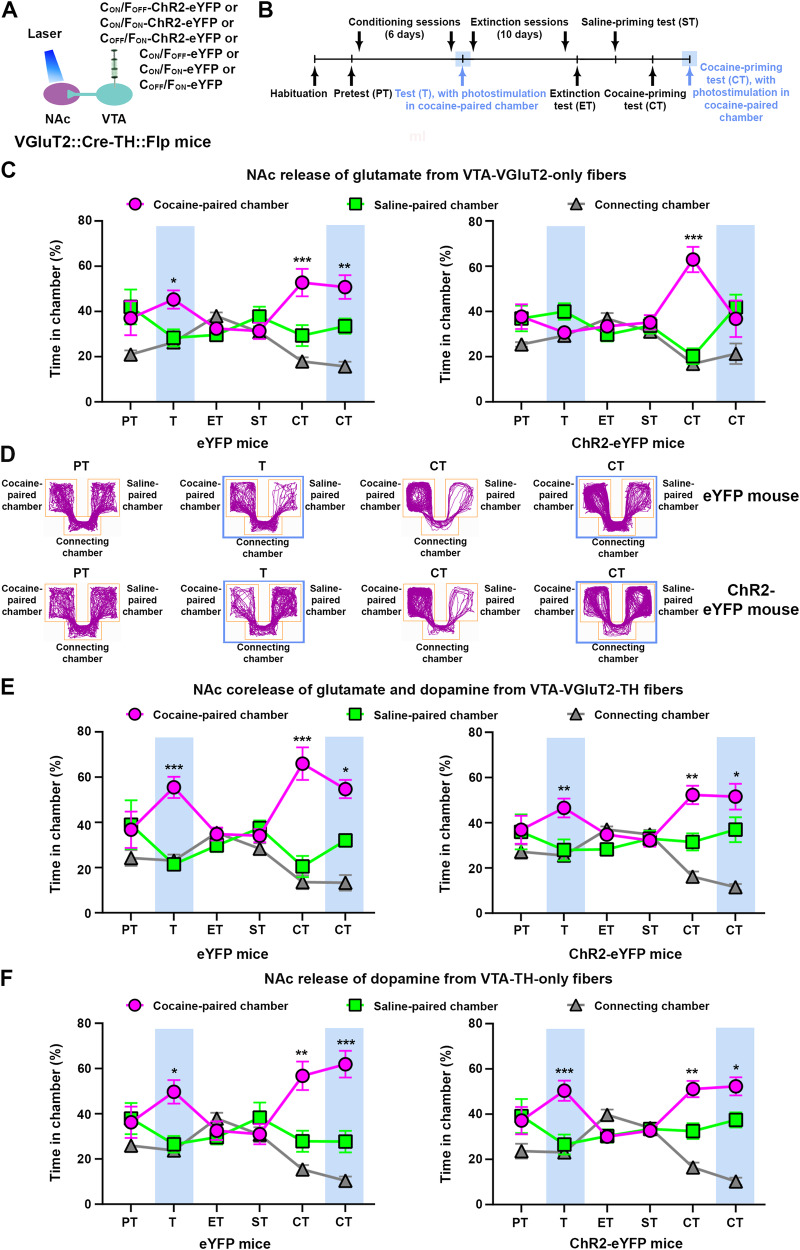
NAc release of glutamate from VTA-VGluT2-only neurons inhibits cocaine priming-induced reinstatement of cocaine CPP. **A** VTA injection of INTRSECT viral vectors and NAc shell optic fibers. **B** Cocaine CPP expression and reinstatement timeline. **C** ChR2-eYFP mice (*n* = 8) spent less time in the cocaine-paired chamber than eYFP control mice (*n* = 8) during an expression test (T) paired with NAc photostimulation of VTA-VGluT2-only fibers. While both groups showed reinstatement of cocaine CPP during a cocaine-priming test (CT) in the absence of photostimulation, only ChR2-eYFP mice avoided the cocaine-paired chamber during a cocaine-priming test (CT) paired with photostimulation (eYFP: chamber x experimental phase: F_10,70_ = 4.17, *p* < 0.001; ChR2-eYFP: F_10,70_ = 5.24, *p* < 0.001, ANOVA with Newman–Keuls post hoc-test). **D** Track plots from an eYFP (top) and a ChR2-eYFP (bottom) mouse in which VTA-VGluT2-only neurons were targeted, during pretest (PT), expression test (T) and cocaine test (CT) in the presence or absence of NAc photostimulation. Blue boxes indicate photostimulation. **E** Both ChR2-eYFP (*n* = 8) and eYFP control mice (*n* = 6) preferred the cocaine-paired chamber during expression (T) and reinstatement (CT) tests paired with or without NAc photostimulation of fibers from dual VTA-VGluT2-TH neurons (eYFP: chamber x experimental phase: F_10,50_ = 5.38, *p* < 0.001; ChR2-eYFP: F_10,70_ = 9.34, *p* < 0.001, ANOVA with Newman–Keuls post-hoc test). **F** Both ChR2-eYFP (*n* = 6) and eYFP control mice (*n* = 8) preferred the cocaine-paired chamber during expression (T) and reinstatement (CT) tests paired with or without NAc photostimulation of VTA-TH-only fibers (eYFP: chamber × experimental phase: F_10,70_ = 7.56, *p* < 0.001; ChR2-eYFP: F_10,50_ = 7.06, *p* < 0.001, ANOVA with Newman–Keuls post-hoc test). *p < 0.05, **p < 0.01, ***p < 0.001, against saline-paired chamber. Light-blue rectangles indicate photostimulation.

Once we established the validity of the VGluT2::Cre-TH::Flpo mice by anatomy, we used these dual recombinase mice to selectively drive the expression of eYFP or ChR2-eYFP in VGluT2-TH, VGluT2-only, or TH-only neurons by intra-VTA injection of INTRSECT vectors. We tested the behavior of the injected mice in the CPP paradigm and evaluated the effects of NAc release of glutamate-only, dopamine-only, or co-release of glutamate and dopamine from VTA fibers in the expression and reinstatement of cocaine-induced CPP (Fig. 3A, B, Supplementary Fig. 8). NAc photostimulation of fibers from VTA-VGluT2-only neurons blocked the expression and reinstatement of cocaine-induced CPP in C_ON_/F_OFF_-ChR2-eYFP mice, but not in control C_ON_/F_OFF_-eYFP mice (Fig. 3C, D). In contrast, NAc release of dopamine-only or co-release of dopamine and glutamate did not affect the expression or reinstatement of cocaine-induced CPP in C_ON_/F_ON_-ChR2-eYFP or C_OFF_/F_ON_-ChR2-eYFP mice (Fig. 3E, F, Supplementary Fig. 9A–D). We conducted NAc electrophysiological recordings in brain slices of mice injected in the VTA with either C_ON_/F_ON_-ChR2-eYFP or C_OFF_/F_ON_-ChR2-eYFP viral vectors to verify the virus functionality. By voltage-clamp recordings, we detected excitatory postsynaptic currents (EPSCs) evoked by NAc photoactivation of dual VTA-VGluT2-TH fibers (Supplementary Fig. 9E, F), whose amplitude was abolished by a cocktail of NMDA and AMPA receptor antagonists and was no further modified by adding a cocktail of D1 and D2 receptor antagonists (Supplementary Fig. 9E, F). Likewise, inward currents evoked by NAc photoactivation of VTA-TH-only fibers were blocked by bath application of a cocktail of D1 and D2 receptor antagonists (Supplementary Fig. 9G, H). These results indicate that the mesoaccumbal glutamatergic pathway mediates the inhibition of both cocaine-induced CPP expression and reinstatement by the release of glutamate from VTA-VGluT2-only neurons, but not by glutamate and dopamine co-release (by dual VTA-VGluT2-TH neurons) or by dopamine release (from VTA-TH-only neurons), which are, nonetheless, capable of evoking EPSCs in the NAc.

### NAc release of glutamate from VTA-VGluT2 fibers inhibits instrumental reinstatement of cocaine-seeking behavior

**Fig. 4:**
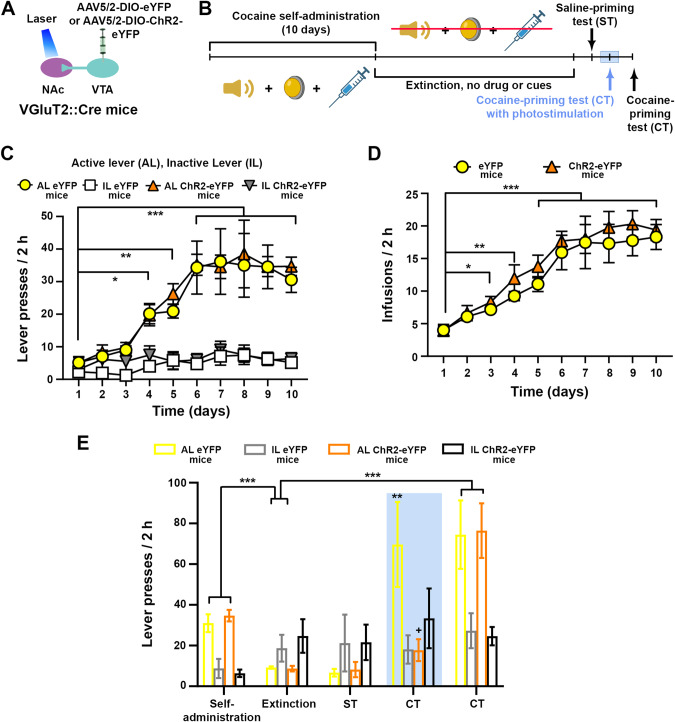
NAc release of glutamate from VTA-VGluT2 fibers inhibits instrumental reinstatement of cocaine-seeking behavior. **A** VTA injection of AAV5/2-DIO-eYFP or AAV5/2-DIO-ChR2-eYFP and NAc shell optic fibers. **B** Cocaine self-administration and reinstatement timeline, showing training with cues and drug, extinction without cues or drug, and reinstatement conditions. Additional extinction sessions were run between each of the reinstatement conditions. **C**, **D** No differences were observed between ChR2-eYFP (*n* = 13) and eYFP control mice (*n* = 13) in the total number of lever presses (**C**, group × day × lever: F_9,216_ = 0.23 *p* = 0.99, ANOVA with Newman–Keuls post-hoc test) or infusions obtained (**D**, group × day: F_9,216_ = 0.29, *p* = 0.98, ANOVA with Newman–Keuls post-hoc test) during self-administration training. *p < 0.05, **p < 0.01, ***p < 0.001, against first day of training. **E** NAc photostimulation of VTA-VGluT2 fibers contingent to presses on the active lever during cocaine-priming test (CT) blocked cocaine-seeking behavior in ChR2-eYFP mice (*n* = 13) when compared to eYFP control mice (*n* = 13, group × day × lever: F_1,24_ = 5.51, *p* < 0.05, ANOVA with Newman–Keuls post-hoc test). Cocaine priming-induced reinstatement of drug-seeking behavior in ChR2-eYFP mice was restored in the absence of VTA photostimulation (day × lever: F_1,24_ = 35.55; *p* < 0.001, ANOVA with Newman–Keuls post-hoc test). **p < 0.01, ***p < 0.001, against extinction. +*p* < 0.05, against eYFP for the same experimental phase. Light-blue rectangle indicates photostimulation.

While the CPP procedure has been and continues to be successfully used to evaluate the reinforcing properties of addictive drugs [23], it has been argued that this short, intermittent, daily access to the drug has its limitations in modeling characteristics of addiction in humans [24]. The reinstatement model established after drug self-administration has become the primary pre-clinical approach for assessing drug motivation [25]. Thus, we determined the extent to which NAc glutamate release from VTA fibers modulates priming-induced reinstatement of cocaine-seeking in mice trained to self-administer cocaine. We trained new cohorts of eYFP and ChR2-eYFP mice (Fig. 4A) daily to press a lever to obtain an intravenous infusion of cocaine (1 mg/kg/infusion). During the training phase, both ChR2-eYFP and eYFP mice learned to associate the pressing of the active lever with cocaine delivery and performed similarly to obtain an equivalent number of infusions (Fig. 4B–D). After 10 days, mice underwent extinction training, in which presses of the active lever no longer resulted in cocaine delivery. The typical “extinction burst” [26] was observed on the first day of extinction (Supplementary Fig. 10A–C). Once the extinction criterion was reached (30% of lever-presses compared to the last self-administration day), we evaluated the effects of saline- or cocaine-priming injections on reinstatement of cocaine-seeking in the presence or absence of NAc photostimulation of VTA-VGluT2 fibers.

After completion of the extinction training, the presses on the active lever were significantly reduced in both eYFP control and ChR2-eYFP mice (Fig. 4E), and without changes after a saline-priming injection (Fig. 4E). As a follow up step, we gave the mice a priming injection of cocaine and paired every press on the active lever with NAc photostimulation of VTA-VGluT2- fibers, detecting reinstatement in eYFP mice but not in ChR2-eYFP mice (Fig. 4E). We next conducted additional extinction sessions, followed by a priming injection of cocaine in the absence of NAc photostimulation, and found that both eYFP and ChR2-eYFP mice reinstated cocaine-seeking (Fig. 4D, Supplementary Fig. 10D). These findings further support our previous results indicating that NAc release of glutamate from VTA-VGluT2 fibers blocked the reinstatement of cocaine-seeking behavior.

### NAc release of glutamate from VTA-VGluT2 fibers inhibits the expression and reinstatement of methamphetamine CPP

Given that cocaine and methamphetamine induce their rewarding effects by increasing the amount of NAc available dopamine [27, 28], and that both induce changes in postsynaptic dopamine receptors of MSNs [29–32], we determined the extent to which the findings observed with cocaine generalize to methamphetamine.

**Fig. 5:**
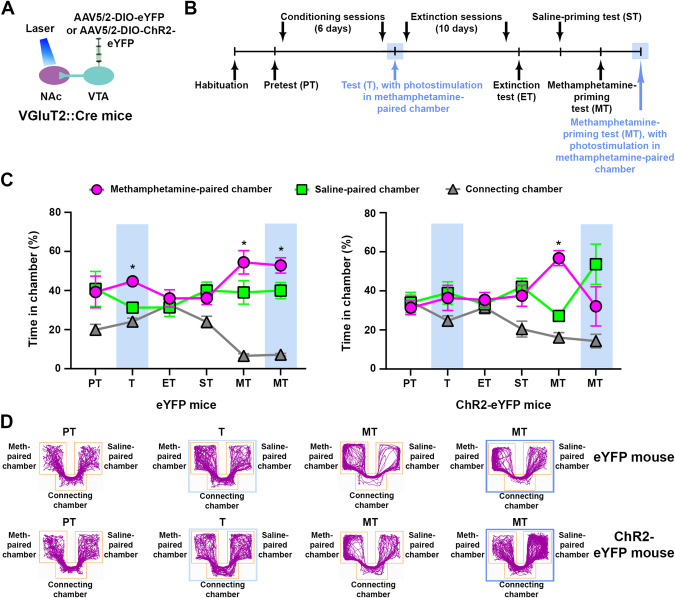
NAc release of glutamate from VTA-VGluT2 fibers inhibits the expression and reinstatement of methamphetamine CPP. **A** VTA injection of AAV5/2-DIO-eYFP or AAV5/2-DIO-ChR2-eYFP and NAc shell optic fibers. **B** Methamphetamine CPP expression and reinstatement timeline. **C** ChR2-eYFP mice (*n* = 6) spent less time in the methamphetamine-paired chamber than eYFP control mice (*n* = 6) during the expression test (T) paired with NAc photostimulation of VTA-VGluT2 fibers. While both groups showed reinstatement of methamphetamine CPP during the methamphetamine-priming test (MT) in the absence of photostimulation, only ChR2-eYFP mice avoided the methamphetamine-paired chamber during the methamphetamine-priming test (MT) paired with photostimulation (eYFP: chamber × experimental phase: F_10,50_ = 4.27, *p* < 0.001; ChR2-eYFP: chamber × experimental phase: F_10,50_ = 4.23, *p* < 0.001, ANOVA with Newman–Keuls post-hoc test). *p < 0.05, against saline-paired chamber. Light-blue rectangles indicate photostimulation. **D** Track plots from an eYFP (top) and a ChR2-eYFP (bottom) mouse during pretest (PT), expression test (T) and methamphetamine test (MT) in the presence or absence of NAc photostimulation. Blue boxes indicate photostimulation.

We prepared eYFP and ChR2-eYFP mice (Fig. 5A, Supplementary Fig. 2G) and trained them in the CPP procedure using methamphetamine (1 mg/kg) as the reinforcer (Fig. 5B). After conditioning sessions, both groups of mice showed preference for the methamphetamine-paired chamber, which was abolished in ChR2-eYFP mice by NAc photostimulation of VTA-VGluT2 fibers (Fig. 5C). Both groups of mice reinstated methamphetamine CPP after a priming injection of methamphetamine in the absence of NAc photostimulation (Fig. 5C). While a follow up methamphetamine-priming injection induced reinstatement in eYFP mice in the presence of NAc photostimulation, it did not induce reinstatement in ChR2-eYFP mice in the presence of NAc photostimulation (Fig. 5C). These results indicate that NAc release of glutamate from VTA-VGluT2 fibers inhibits the expression and reinstatement of CPP for different types of psychostimulants.

## Discussion

The involvement of the mesoaccumbal dopamine pathway in the reinforcing effects of psychostimulants has been demonstrated by lesion studies showing that destruction of dopamine neurons within the VTA disrupts cocaine-self administration [4]; similar results were observed after depletion of dopamine from the NAc [33]. In addition to dopamine, glutamate neurotransmission within the NAc from different brain areas has been implicated in the modulation of drug-seeking behaviors; these brain areas include the medial PFC, basolateral amygdala, ventral hippocampus, ventral subiculum, and paraventricular nucleus of the thalamus, which all send glutamatergic afferents to the NAc [14, 34, 35]. In contrast, we demonstrated that NAc mShell release of glutamate resulting from activation of VTA glutamatergic fibers did not affect the acquisition of cocaine-induced CPP, but it blocked the expression, priming- and stress-induced reinstatement of cocaine CPP and the priming-induced reinstatement of cocaine-seeking in the self-administration procedure. We further extended these observations by demonstrating that NAc mShell release of glutamate from VTA glutamatergic fibers blocked the expression and priming-induced reinstatement of methamphetamine CPP. Collectively, these data indicate that (1) in addition to the well-recognized mesoaccumbal dopamine system known to mediate psychostimulant-seeking behavior, there is a parallel mesoaccumbal glutamatergic system that plays a role in inhibiting psychostimulant preference and seeking and (2) that different sources of glutamate inputs to the NAc are involved in different behaviors associated with cocaine exposure (Supplementary Fig. 11).

We previously demonstrated that activation of mesoaccumbal glutamatergic inputs induces the depolarization of a subpopulation of NAc PV-GABA interneurons, which are preferentially innervated by VTA glutamate neurons, and that their depolarization promotes a general inhibition of the MSNs’ output [16]. Electrophysiological recordings have shown that cocaine or amphetamine administration increases the excitability of PV interneurons [36, 37], and the selective silencing of NAc PV interneurons inhibited amphetamine-induced CPP, and locomotor sensitization to repeated amphetamine administration [38], indicating that PV interneurons are required for the behavioral responses to amphetamine. In addition, other studies have shown that dopaminergic agents modulate the firing activity of PV interneurons, with agonists increasing and antagonists decreasing their firing rate [36, 39], an effect that appears to be mediated by postsynaptic D1 and presynaptic D2 dopamine receptors [39]. Therefore, dopamine release from VTA neurons exerts a strong regulation on the NAc output via direct action on MSNs and indirect action on PV interneurons. Given our previous demonstration that NAc mShell release of glutamate induces cFos expression preferentially in PV-GABA interneurons [16], we propose that a parallel mesoaccumbal glutamatergic pathway also plays a role in the modulation of psychostimulant-seeking behavior by the selective activation of a subset of PV-GABA interneurons. Recent electrophysiological studies found that activation of a basolateral amygdala glutamatergic projection to the NAc shell induced a rapid fast-spiking interneuron (putative PV interneurons)-mediated inhibition of MSNs, which promoted the acquisition of cocaine self-administration [40]. This suggests that the source of glutamatergic innervation to PV interneurons of the NAc may differentially regulate psychostimulant-seeking behavior, as recently proposed for cocaine [41].

In conclusion, our findings indicate that activation of the VTA-NAc glutamatergic pathway decreases psychostimulant preference and seeking and underscore the contribution of diverse sources of glutamate inputs to the NAc to either increase or decrease drug-seeking behavior (Supplementary Fig. 11). We propose that, in addition to the mesoaccumbal dopamine pathway known to play a role in the rewarding and reinforcing effects of psychostimulants, there is a parallel mesoaccumbal glutamatergic pathway that plays a role in suppressing the expression and reinstatement of psychostimulant preference and seeking.

## Supplementary information

Supplementary Information

## Source data

Source data

## Data Availability

The datasets generated and/or analyzed during the current study are available at the Zenodo repository (DOI 10.5281/zenodo.11624452).
