# Supplementary material for: VTA glutamatergic projections to the nucleus accumbens suppress psychostimulant-seeking behavior
Source: Neuropsychopharmacology. 2024 Jun 26;49(12):1905–15. doi: 10.1038/s41386-024-01905-3 (PMC11473768; doi:10.1038/s41386-024-01905-3)
Supplement: Supplementary file 1 — Supplementary Information [file 41386_2024_1905_MOESM1_ESM.pdf]

Neuropsychopharmacology

Supplementary information

VTA glutamatergic projections to the nucleus accumbens suppress psychostimulant-seeking behavior

M. Flavia Barbano, PhD; Jia Qi, PhD; Emma Chen, BS; Uzma Mohammad, BA; Orlando Espinoza, BS; Marcos Candido, BS; Huiling Wang, MD/PhD; Bing Liu, MD; Suyun Hahn, PhD; François Vautier, PhD; Marisela Morales, PhD

Supplementary Materials and Methods

Subjects. Male and female VGluT2::IRES::Cre (Slc17a6tm2(cre)Lowl/J, in C57BL/6J background from the Jackson Laboratories, Bar Harbor, ME), and TH::Flpo x VGluT2::IRES::Cre mice (cross between VGluT2::IRES::Cre and TH-2A-Flpo mice [C57BL/6N-Thtm1Awar/Mmmh, in C57BL/6J background from the Mutant Mouse Resource and Research Centers, Davis, CA]) were bred in the NIDA/IRP animal facility (Baltimore, MD) for at least five generations and were used in behavioral and anatomical experiments. Groups of 2-5 mice (weighing 20-30 g at the start of the experiments) were housed in an animal vivarium maintained on a direct 12-h light-dark cycle (lights on at 7:00 am) and at a constant temperature of 23°C. All testing occurred during the light part of the light-dark cycle. Animals were kept undisturbed at least one week before the start of any experimental procedure and were handled and weighed daily to minimize handling stress during experiments. Food and water were provided *ad libitum* except during experimental sessions, unless otherwise stated. Littermates of the same sex were randomly assigned to experimental groups. Animal care and use were in strict accordance with institutional and international standards and were approved by the National Institute on Drug Abuse Animal Care and Use Committee (ASP: 21-INRB-2).

Surgeries. Intra-cranial surgeries. Each mouse was anesthetized with isofluorane (2-2.5% for induction, 1–2% for maintenance; Butler Schein, Owings Mills, MD), was placed in a stereotaxic frame (David Kopf Instruments, Tujunga, CA) and its skull was exposed and leveled. Cre-inducible adeno-associated virus (AAV, serotypes 2 or 5; 1.5-3 × 10^{12} genomes/ml, UNC Vector Core Facility, Chapel Hill, NC) coding for the light-sensitive protein Channelrhodopsin-2 (ChR2) tethered to the enhanced yellow fluorescent protein (eYFP) or coding for eYFP alone under control of the EF1α promoter were employed. INTRSECT (INTronic Recombinase Sites Enabling Combinatorial Targeting) viral vectors coding for ChR2-eYFP or for eYFP alone under control of the EF1α promoter were also employed (Stanford University Gene Vector and Virus Core, Stanford, CA). Briefly, 200-250 nl of AAV1-EF1α-DIO-hChR2(H134R)-eYFP (corresponding ChR2-eYFP mice), AAV1-EF1α-DIO-eYFP (corresponding control eYFP mice), AAV-DJ-hSyn-Cre_{ON}/Flp_{ON}-ChR2-eYFP (corresponding Con/Fon-ChR2-eYFP mice), AAV-DJ-hSyn-Cre_{ON}/Flp_{ON}-eYFP (corresponding control Con/Fon-eYFP mice), AAV-DJ-hSyn-Cre^{ON}/Flp^{OFF}-ChR2-eYFP (corresponding Con/Foff-ChR2-eYFP mice), AAV-DJ-hSyn-Cre_{ON}/Flp_{OFF}-eYFP (corresponding control Con/Foff-eYFP mice), AAV-DJ-hSyn-Cre_{OFF}/Flp_{ON}-ChR2-eYFP (corresponding Coff/Fon-ChR2-eYFP mice) or AAV-DJ-hSyn-Cre_{OFF}/Flp_{ON}-eYFP (corresponding control Coff/Fon-eYFP mice) were injected into the midline of the VTA (AP: −3.2, ML: ±0, DV: −4.3, from bregma). Injections were done at a flow rate of 100 nl/min, using an UltraMicroPump with Micro 4 controller (WPI Inc., Sarasota, FL), 5 µl syringes, and 33 g needles (Hamilton, Reno, NV). The needle was left in place for additional 3 min to prevent reflux. Mice received bilateral intracranial optical fibers (200 µm diameter, BFL37–200, Thorlabs, Newton, NJ) directed to the NAc medial shell (mShell; AP +1.4, ML ±1.3, DV -4.0, at a 10º angle) eight weeks after viral injections. One stainless-steel screw and dental acrylic cement were used to anchor the optic fibers to the skull. In all surgical procedures, animals were given the analgesic meloxicam (5 mg/kg; Covetrus, Dublin, OH) to prevent post-surgical pain or discomfort and were allowed at least 10 days of recovery before the beginning of any experimental manipulation. Body weight was measured daily after surgery to monitor for proper recovery.

Intra-venous surgeries. Mice were anesthetized with isoflurane (2-2.5% for induction, 1–2% for maintenance; Butler Schein) and then implanted with indwelling i.v. silastic catheters. Briefly, a mouse i.v. catheter tubing (MSB-SA, SAI Infusion Technologies, Lake Villa, IL) was inserted 1.3 cm into the right jugular vein and anchored with suture. The remaining tubing was run subcutaneously to exit at the midscapular region. After surgery, mice were allowed to recover for at least 3 days prior to the initiation of self-administration sessions. The catheter was flushed daily with 0.1 ml of a saline solution containing heparin (30 U/ml) and gentamicin (4.8 mg/ml). The patency of the intravenous catheters was evaluated at the end of the self-administration training sessions by infusion of 0.1 ml etomidate (2 mg/ml, Hospira, Inc., Lake Forest, IL) through the catheter. If prominent signs of anesthesia were not apparent within 3 s of the infusion, the mouse was removed from the experiment.

RNAscope *in situ* hybridization combined with immunolabeling. Eight weeks after viral injections, mice were deeply anesthetized with chloral hydrate (0.5 ml/kg) and perfused transcardially with 4% (w/v) paraformaldehyde (PF) in 0.1 M phosphate buffer (PB), pH 7.3. Brains were left in 4% PF for 2 h and transferred to 18% sucrose in DEPC-treated PB overnight at 4°C. Free-floating coronal serial VTA cryosections (16 µm) were prepared and were incubated for 2 h at 30°C with mouse anti-GFP antibody (1:500, 632381, Takara, Mountain View, CA) in DEPC-treated PB with 0.5% Triton X-

100 supplemented with RNasin (Promega, Madison, WI) to phenotype VGluT2-only, TH-only or dual VGluT2-TH neurons expressing eYFP. Sections were rinsed 3 x 10 min with DEPC-treated PB and incubated with secondary donkey anti-mouse Alexa Fluor 488 (1:100, 715-545-151 Jackson Immunoresearch, West Grove, PA) for 1 h at 30°C. Sections were then rinsed with DEPC-treated PB, mounted onto Fisher SuperFrost slides and dried overnight at 60°C. RNAscope *in situ* hybridization was then performed according to the manufacturer's instructions. Briefly, sections were treated with heat and protease digestion followed by hybridization with a cocktail of probes for detection of transcripts encoding VGluT2 mRNAs (319171, Advanced Cell Diagnostics, Newark, CA) and TH mRNAs (317621-C2, Advanced Cell Diagnostics). Additional sections were hybridized with the bacterial gene DapB as a negative control, which did not exhibit fluorescent labeling. VGluT2 and TH were detected by Atto 550 and Atto 647. RNAscope *in situ* hybridization and immunolabeled sections were viewed, analyzed, and photographed with a Zeiss LSM880 confocal microscope equipped with Airyscan/CY7.5 (Zeiss, White Plains, NY). Negative control hybridizations showed negligible fluorophore expression. Neurons were counted when the stained cell was at least 5 µm in diameter. Pictures were adjusted to match contrast and brightness by using Adobe Photoshop (Adobe Systems Inc., San Jose, CA). The number of mice analyzed (n=3-4) was based on previous studies in our lab using radioactive detection of VGluT2 mRNA from rat VTA neurons[15,42]. Five sections, covering bregmas -2.92 mm to -3.88 mm, were used per mouse.

Histological verification. After behavioral testing, all mice were deeply anesthetized and brain tissue for immunodetection of eYFP was processed as follows: free-floating coronal sections (30 µm) were incubated for 1 h in PB supplemented with 4% BSA and 0.3% Triton X-100. Sections were then incubated with primary antibody: mouse anti-GFP antibody (1:500, 632381, Takara), overnight at 4°C. After rinsing 3 × 10 min in PB and incubation in biotinylated goat anti-mouse antibody (1:200, Vector Laboratories, Burlingame, CA) for 2 h at room temperature (RT), the sections were rinsed with PB and incubated for 1 h at room temperature in avidin-biotinylated horseradish peroxidase (1:200, ABC kit, Vector Laboratories). Sections were rinsed, and the peroxidase reaction was developed with 0.05% 3,3'-diaminobenzidine (DAB) and 0.03% H_2O_2. Sections were mounted on gelatin-coated slides. Bright field images were collected with an Olympus MVX10 with 0.63× objective (Evident, Waltham, MA) or an Olympus VS200 Scanner with 4x objective (Evident, Waltham, MA). For fluorescence immunodetection of eYFP and tyrosine hydroxylase (TH), free-floating coronal sections were incubated with cocktails of primary antibodies: sheep anti-TH antibody (1:1000, AB1542, Millipore, Billerica, MA) + mouse anti-GFP, overnight at 4°C. After rinsing 3 × 10 min in PB, sections were incubated in a cocktail of the corresponding fluorescence secondary antibodies (1:100, Alexa Fluor-488 or Alexa Fluor-594, Jackson Immunoresearch Laboratories) for 2 h at room temperature. After rinsing, the sections were mounted on slides and coverslipped using antifading mounting medium with DAPI (Vectashield, Vector Laboratories). Fluorescent images were collected with a Zeiss LSM880 confocal microscope equipped with Airyscan/CY7.5 (Zeiss). Images were taken sequentially with different lasers with 5× (for low magnification) or 20× (for high magnification) objectives.

Drugs. Cocaine hydrochloride and methamphetamine hydrochloride were obtained from the National Institute on Drug Abuse (NIDA) Drug Supply Program through the NIDA Intramural Research Program pharmacy and were dissolved in sterile 0.9% physiological saline. We used 5-15 mg/kg administered intraperitoneally (i.p.) for the cocaine-induced CPP training, 1 mg/kg per infusion for the self-administration training, and 15 mg/kg i.p. for cocaine-priming injections. We used 1 mg/kg administered i.p. for methamphetamine-induced CPP training and priming injections.

Ex vivo electrophysiology. We prepared Con/Fon-ChR2-eYFP (n = 6) and Coff/Fon-ChR2-eYFP (n = 6) mice by injecting the corresponding INTRSECT viral vector into the VTA. Eight weeks after injections, mice were deeply anesthetized with isoflurane, were decapitated under isoflurane anesthesia and their brains were quickly removed into oxygenated (95% O2/5% CO2), ice-cold high sucrose-based cutting solution (in mM): 220 sucrose, 2.5 KCl, 0.5 $CaCl_2$, 7 $MgSO_4$, 1.25 NaH_2PO_4, 26 $NaHCO_3$, 20 glucose (pH 7.2-7.4). Coronal slices containing the NAc (220 µm) were cut using a vibratome (VT1200, Leica, Nussloch, Germany), which were transferred into an oxygenated N-Methyl-D-glucamine (NMDG)-based recovery solution at 33°C for 7 min (in mM): 93 NMDG, 3 KCl, 10 $MgSO_4$, 0.5 $CaCl_2$, 1.2 NaH_2PO_4, 30 $NaHCO_3$, 25 glucose, 20 HEPES, 5 sodium ascorbate, 3 sodium pyruvate (pH 7.3-7.4, ~310 mOsm-1). After recovery, slices were incubated for more than 30 min at room temperature before recordings in oxygenated artificial cerebrospinal fluid (aCSF) containing (in mM): 126 NaCl, 3 KCl, 2.4 $CaCl_2$, 1.2 NaH_2PO_4, 26 $NaHCO_3$, 11 glucose, $MgCl_2$ (pH 7.3-7.4, ~320 mOsm-1). For electrophysiological recordings, slices were transferred to a recording chamber continuously perfused with fully oxygenated aCSF at 33°C. Patch pipettes (4-6 MΩ) were pulled from filamented borosilicate glass capillaries (World Precision Instruments, Sarasota, FL) with a PC-100 micropipette puller (Narishige, Tokyo, Japan) and backfilled with an

internal solution containing (in mM): 140 potassium gluconate, 2 NaCl, 1.5 $MgCl_2$, 10 HEPES, 4 Mg-ATP, 0.3 Na_2-GTP, 10 Tris-phosphocreatine, 0.1 ethylene glycol-bis (2-aminoethyl ether)-N,N,N,N'-tetraacetic acid (EGTA) with 0.08-0.1 % biocytin (pH 7.2, 280-290 mOsm-1). Cells were visualized on an upright microscope using infrared differential interference contrast video microscopy. Whole-cell voltage-clamp recordings were performed using a MultiClamp 700B amplifier (Molecular Devices, Sunnyvale, CA), low-pass filtered at 2 kHz and digitized at 10 kHz with pClamp 11.2 software (Molecular Devices). Only postsynaptic NAc neurons surrounded by eYFP fibers were selected to measure optical-evoked currents (held at - 80 mV, 9 neurons for each phenotype recorded). NAc photostimulation of both TH-only and dual VGluT2-TH fibers from the VTA resulted in inward currents that were obtained with single-pulses of 473 nm wavelength blue light (5 ms). To determine whether the excitatory postsynaptic currents (EPSCs) observed in Con/Fon-ChR2-eYFP mice were evoked by glutamate and dopamine release, a cocktail of the NMDA (D-AP5, 50 µM) and the AMPA (CNQX, 10 µM) receptor antagonists followed by a cocktail of the D1 (SCH-23390, 10 µM) and the D2 (sulpiride, 10 µM) receptor antagonists were bath-applied. In the case of Coff/Fon-ChR2-eYFP mice, a cocktail of the D1 (SCH-23390, 10 µM) and the D2 (sulpiride, 10 µM) receptor antagonists was added to aCSF during recordings of postsynaptic inward currents. Current amplitudes were measured by the differential between peak amplitudes and baselines.

Apparatus. _Conditioned place preference._ A three-chamber place conditioning apparatus was used for the behavioral experiments (ANY-box, Stoelting, Wood Dale, IL), which consisted of two main chambers (20 × 18 × 35 cm) and a connecting chamber (20 × 10 × 35 cm) with distinct wall patterns and a non-reflective base plate. The position of the animal was monitored via an overhead closed-circuit camera interfaced with video tracking software (ANY-maze, Stoelting).

Operant behavior. Drug self-administration training and testing occurred in operant chambers (Model ENV-307W-C, MED Associates, Inc., St. Albans, VT) equipped with two retractable levers. One of them was selected as the reinforced lever for delivering the drug and the other was selected as the non-reinforced lever. Active pressing on the reinforced lever resulted in a cocaine infusion while pressing on the non-reinforced lever did not result in cocaine infusion. Chambers were made of aluminum and clear acrylic, had grid floors, and were housed in sound- and light-attenuating boxes equipped with fans to provide ventilation and ambient noise. A stimulus light, located above the reinforced lever, and a 75 dB tone (2,000 Hz) were paired contingently with the delivery of the drug. When mice responded on the reinforced lever, the stimulus light and the tone went on, and a drug infusion was delivered. Cocaine was infused via a syringe that was mounted on a microinfusion pump (PHM-100 MED Associates, Inc.) and connected via Tygon tubing (AAD04103, Saint Gobain Performance Plastics, Malvern, PA) to a single channel liquid swivel (375/22PS, Instech Laboratories, Plymouth Meeting, PA) and to the mouse intravenous (i.v.) catheters. The swivel was mounted on a counterbalanced arm above the operant chamber.

Behavioral studies. _Acquisition of cocaine-induced conditioned place preference (CPP)._ Mice were placed in the connecting chamber with free access to both main chambers for 15 min on habituation and pretest days. Fiber-optic cables were attached via FC/PC connector to 473 nm lasers (OEM/Opto Engine LLC, Midvale, UT) for photostimulation. During the pretest, mice were connected to the fiber-optic cables, but the lasers were off. To test the effects of NAc photostimulation on the acquisition of cocaine-induced CPP, mice were conditioned during 6 daily sessions with a non-biased design (cocaine injections were counterbalanced across the chambers and the time of the day). Each daily session included one cocaine-paired and one saline-paired conditioning training trial that lasted 30 min and that was administered in the morning or in the afternoon. During cocaine trials, mice were administered with cocaine (15 mg/kg, i.p.) before being confined to the cocaine-paired chamber, in which they received NAc photostimulation (473 nm, ~5mW, 20 Hz, 10 ms duration, 5 sec on/off) for the duration of the trial. During saline trials, mice were administered with saline before being confined to the saline-paired chamber, without photostimulation. After the 6th daily training session, on the CPP test day, mice had free access to all three chambers for 15 min without NAc photostimulation. Time spent in each chamber during the tests was recorded for each mouse.

Expression of cocaine- or methamphetamine-induced CPP. To test the effects of NAc photostimulation on the expression of cocaine- or methamphetamine-induced CPP, mice were trained during 6 daily sessions as described for the acquisition of the cocaine-induced CPP experiment, but no NAc photostimulation was administered during the conditioning training trials. On the CPP test day, mice had access to each of the three chambers for 15 min while receiving trains of bilateral NAc photostimulation (473 nm, ~5 mW, 20 Hz, 10 ms duration) each time they entered, and for as long as they remained

in the cocaine- or methamphetamine-paired chamber. No photostimulation was administered upon entry to the saline-paired chamber. Time spent in each chamber during testing was recorded, and the CPP test was followed by 10 sessions of extinction training. On the extinction test day, mice were allowed free access to all the chambers for 15 min. A saline-priming test (15 min) was performed 24 h after the extinction test to confirm that there was no stress-induced reinstatement caused by the injection. Cocaine- or methamphetamine-priming tests (15 min) were performed 24 h after the saline-priming test, and mice received cocaine (15 mg/kg, i.p.) or methamphetamine (1 mg/kg, i.p.) instead of saline injections.

Reinstatement of cocaine- or methamphetamine-induced CPP. To test the effects of NAc photostimulation on the reinstatement of cocaine- or methamphetamine-induced CPP, mice were trained during 6 daily sessions as described for the acquisition of cocaine-induced CPP experiment but no NAc photostimulation was administered during the conditioning training trials. On the CPP test day, no NAc photostimulation was administered and mice had access to explore the three chambers for 15 min. Then, mice underwent 10 sessions of extinction training and, on the extinction test day, they were allowed free access to all the chambers for 15 min. A saline-priming test (15 min) was performed 24 h after the extinction test and the cocaine-priming test (15 min) was performed 24 h after the saline-priming test, where mice received a cocaine injection (15 mg/kg, i.p.) and had access to each of the three chambers for 15 min while receiving trains of bilateral NAc photostimulation (473 nm, ~5 mW, 20 Hz, 10 ms duration) each time they entered, and for as long as they remained in the cocaine-paired chamber. After conducting additional extinction sessions, a new cocaine-priming test was conducted in which no NAc photostimulation was administered. In another experiment, during saline-priming test day, mice had free access to each chamber for 15 min while receiving trains of bilateral NAc photostimulation (473 nm, ~5 mW, 20 Hz, 10 ms duration) each time they entered, and for as long as they remained in the saline-paired chamber. Time spent in each chamber was recorded for all the tests performed. A methamphetamine-priming injection (1 mg/kg, i.p.) was administered instead for reinstatement of methamphetamine-induced CPP experiments.

Stress-induced reinstatement of cocaine CPP. To test the effects of NAc photostimulation on stress-induced reinstatement of cocaine CPP, mice were trained during 6 daily sessions as described for the acquisition of cocaine-induced CPP experiment without NAc photostimulation administered during the conditioning training trials. On the CPP test day, no NAc photostimulation was administered and mice had access to explore the three chambers for 15 min. Then, mice underwent 10 sessions of extinction training, and, on the extinction test day, they were allowed free access to all the chambers for 15 min. On the next day, mice received a total of 15 brief foot-shocks (0.5 s, 0.8 mA) over the course of a 15-minute session with variable inter-shock intervals (10-90 s, average 57 s) and were immediately placed in the CPP apparatus, where they had access to each of the three chambers for 15 min while receiving trains of bilateral NAc photostimulation (473 nm, ~5 mW, 20 Hz, 10 ms duration) each time they entered, and for as long as they remained in the cocaine-paired chamber.

Photostimulation-induced place avoidance. The place avoidance experiment was divided into 2 phases: pretest and test. During the 15-min pretest phase, ChR2-eYFP and eYFP mice were connected to the fiber-optic cable and laser, placed in the connecting chamber, and allowed to freely explore the entire CPP apparatus. Time spent in each compartment was measured. In the test phase, one chamber (counterbalanced across mice) was selected as the laser-paired chamber: entrance to this chamber by the mouse triggered continuous trains of NAc photostimulation (473 nm, ~5 mW, 20 Hz, 10 ms duration). The photostimulation remained on as long as the mouse was within the chamber. Entrance to the other chamber was without consequences. Each test lasted 30 min and was repeated for 4 days.

Drug self-administration training. Self-administration responding was maintained by cocaine (1 mg/kg/injection) delivered in 30.625 µl over 2.5 s. Daily self-administration sessions lasted 120 min and were conducted 7 days per week and were initiated by the presentation of both the reinforced and non-reinforced levers. The house light was on during the entire session. Presses on the reinforced lever led to cocaine infusions and the presentation of a burst of white noise and the light cue (located above the reinforced lever) for 2.5 s. Mice were trained to lever press for cocaine under a fixed ratio 1 (FR1) schedule of reinforcement. A 20 s time-out period was established after the mice obtained each cocaine injection; during this 20 s period, the white noise was off, the cue light was on, and no drug infusions were delivered upon responses on the reinforced lever. All the responses performed on the reinforced and non-reinforced levers during the 20 s time-out were recorded. The criteria for the acquisition was achieved when mice maintained a 75% of responses on the reinforced lever and a minimum of 10 reinforcements per session. The training sessions were

conducted over 10 days after which the catheter patency was evaluated. Only mice with a patent catheter at the end of the training sessions moved on to the extinction sessions.

Drug self-administration extinction. During the extinction sessions, presses on the reinforced lever resulted in the activation of the pump to maintain the usual experimental environment but animals did not receive drug infusions. Also, the light cue and white noise were off during this period. Mice were given 2 h daily extinction sessions conducted 7 days per week until they made responses on the reinforced lever equal to or less than 30% of the responses performed during the last day of the cocaine training sessions. After achieving the extinction criteria, the reinstatement sessions started. Additional extinction sessions were run between reinstatement sessions.

Drug self-administration reinstatement. We tested three different experimental conditions to induce reinstatement of cocaine-seeking behavior: a session with a priming injection of saline, a session with a priming injection of cocaine (15 mg/kg) administered i.p. in which no photostimulation was administered, and a session with a priming injection of cocaine (15 mg/kg) administered i.p. in which lever presses on the reinforced lever were concomitant with photostimulation (473 nm, ~5mW, 20 Hz, 10 ms duration, 5 s). The order of the sessions was counterbalanced across the mice. Mice received an injection of saline or cocaine and were immediately confined to the self-administration boxes to start the reinstatement test. No light cue or white noise were associated with the reinforced lever pressing to avoid a cue-induced reinstatement contribution to the procedure.

Quantification and statistical analysis. The number of mice ("n") used in each group or condition is described either in the main text or in the legend of each figure. No statistical methods were used to predetermine sample size, but sample sizes are consistent with those reported in previous publications in the field and in our laboratory. In optogenetic experiments, mice with fiber tip placement outside of the targeted structure were excluded from the analysis. All attempts at replication were successful. Data analysis of results obtained from each experimental procedure was conducted in a blind manner. Results are presented as mean ± SEM, with behavioral data analyzed using a multifactorial analysis of variance (MANOVA) with group (eYFP or ChR2-eYFP) as the between-subjects factor, and days, trials, or phases of testing as within-subject factors. When the same mice were tested under different conditions, a repeated measures ANOVA was used instead. For significant overall interactions, further analyses of partial interactions were carried out. Post-hoc analyses were performed using the Newman-Keuls test when the initial p value was significant. A result was considered significant if $p < 0.05$. All data were analyzed using Statistica software (Statsoft Inc., Tulsa, OK).

Additional references:

42- Root DH, Mejias-Aponte CA, Zhang S, Wang HL, Hoffman AF, Lupica CR et al., Single rodent mesohabenular axons release glutamate and GABA. *Nat Neurosci* 2014; **17**: 1543-1551.

Supplementary figure legends

Supplementary figure 1. VTA detection of viral injection sites. A. VTA injection of AAV5/2-DIO-eYFP or AAV5/2-DIO-ChR2-eYFP and NAc shell optic fibers. **B-C.** Maximal (blue) and minimal (red) rostro-caudal extension of viral injections (**B**, eYFP; **C**, ChR2-eYFP) within the VTA. **D-E.** VTA immunofluorescence detection of VGluT2-expressing neurons (green) and TH (red) for an eYFP control mouse (**D**) and a ChR2-eYFP mouse (**E**). IF, interfascicular nucleus; ml, medial lemniscus; PBP, parabrachial pigmented nucleus; PN, paranigral nucleus; RLi, rostro linear nucleus; VTA, ventral tegmental area; vtgx, ventral tegmental decussation.

Supplementary figure 2. NAc location of optical probes. A. VTA injection of AAV5/2-DIO-eYFP or AAV5/2-DIO-ChR2-eYFP and NAc shell optic fibers. **B-E.** Low (**B**, **D**) and high (**C**, **E**) magnification NAc images showing eYFP fibers (green) from VTA-VGluT2 neurons and optic fiber placements for an eYFP control mouse (**B-C**) and a ChR2-eYFP mouse (**D-E**). **F.** NAc optic fiber placements in eYFP (left) and ChR2-eYFP (right) mice for figure 1 and supplementary figure 4. **G.** NAc optic fiber placements in eYFP (left) and ChR2-eYFP (right) mice for figures 2, 4, 5, supplementary figures 5 and 10. **H.** NAc optic fiber placements in eYFP (left) and ChR2-eYFP (right) mice for supplementary figure 6. Core, nucleus accumbens core; CPU, caudate putamen nucleus; cp, cerebral peduncle; mShell, nucleus accumbens medial shell.

Supplementary figure 3. Track plots for acquisition, expression, and priming-induced reinstatement experiments. A. VTA injection of AAV5/2-DIO-eYFP or AAV5/2-DIO-ChR2-eYFP and NAc shell optic fibers. **B.** Cocaine CPP acquisition timeline. **C.** Track plots from an eYFP (left) and a ChR2-eYFP (right) mouse during pretest (PT) and expression test (T) after administration of NAc photostimulation during the conditioning sessions. **D.** Cocaine CPP expression timeline. **E.** Track plots from an eYFP (top) and a ChR2-eYFP (bottom) mouse during pretest (PT), expression test (T), and cocaine test (CT) in the presence or absence of NAc photostimulation. Blue boxes indicate photostimulation. **F.** Cocaine CPP reinstatement timeline. **G.** Track plots from an eYFP (top) and a ChR2-eYFP (bottom) mouse during pretest (PT), expression test (T), and cocaine test (CT) in the presence or absence of NAc photostimulation. Blue boxes indicate photostimulation.

Supplementary figure 4. NAc release of glutamate from VTA-VGluT2 fibers induces aversion but does not modify the acquisition of cocaine CPP in the same cohort of mice. A. VTA injection of AAV5/2-DIO-eYFP or AAV5/2-DIO-ChR2-eYFP and NAc shell optic fibers. **B.** Laser-induced place aversion timeline. **C.** ChR2-eYFP mice (n = 6) spent significantly less time in the laser-paired chamber than eYFP control mice (n = 7) during and after NAc photostimulation sessions (eYFP: chamber x experimental phase: $F_{10,60}$=0.33, p=0.97, n.s.; ChR2-eYFP: chamber x experimental phase: $F_{10,50}$=2.98, p<0.01, ANOVA with Newman-Keuls post-hoc test). * p<0.05, ** p<0.01, *** p<0.001, against laser-unpaired chamber. **D.** Cocaine CPP acquisition timeline for the same mice from the laser-induced place aversion experiment. **E.** Both eYFP (n=7) and ChR2-eYFP mice (n=6) spent more time in the cocaine-paired chamber after the conditioning sessions (eYFP: chamber x experimental phase: $F_{2,12}$=7.56, p<0.01; ChR2-eYFP: $F_{2,10}$=6.26, p<0.05, ANOVA with Newman-Keuls post-hoc test). ** p<0.01, *** p<0.001, against saline-paired chamber. Light-blue rectangles indicate photostimulation.

Supplementary figure 5. NAc shell release of glutamate from VTA-VGluT2 fibers inhibited the expression and reinstatement of cocaine CPP in female mice. A. VTA injection of AAV5/2-DIO-eYFP or AAV5/2-DIO-ChR2-eYFP and NAc shell optic fibers. **B.** Cocaine CPP expression and reinstatement timeline. **C.** ChR2-eYFP female mice (n=8) spent less time in the cocaine-paired chamber than eYFP female mice (n=6) during the expression test (T) paired with NAc photostimulation of VTA-VGluT2 fibers. While both groups showed reinstatement of cocaine CPP during the cocaine-priming test (CT) in the absence of photostimulation, only ChR2-eYFP female mice avoided the cocaine-paired chamber during the cocaine-priming test (CT) paired with photostimulation (eYFP: chamber x experimental phase: $F_{10,50}$=2.86, p<0.01; ChR2-eYFP: $F_{10,70}$=4.40, p<0.001, ANOVA with Newman-Keuls post-hoc test). * p<0.05, ** p<0.01, against saline-paired chamber. Light-blue rectangles indicate photostimulation.

Supplementary figure 6. Suppression of cocaine-induced CPP expression and reinstatement by NAc release of glutamate from VTA-VGluT2 fibers does not depend on the dose of cocaine used. A. VTA injection of AAV5/2-DIO-eYFP or AAV5/2-DIO-ChR2-eYFP and NAc shell optic fibers. **B.** Cocaine CPP expression and reinstatement timeline. **C-D.** ChR2-eYFP mice injected with doses of 5 mg/kg (**C**, n=7) or 10 mg/kg (**D**, n=8) of cocaine spent less time in the cocaine-paired chamber than eYFP control mice (**C**, n=7; **D**, n=6) during the expression test (T) paired with NAc photostimulation of VTA-VGluT2 fibers. While both groups showed reinstatement of cocaine CPP during the cocaine-priming test (CT) in the absence of photostimulation, only ChR2-eYFP mice avoided the cocaine-paired chamber during the cocaine-priming test

(CT) paired with photostimulation (**C**, eYFP: chamber x experimental phase: $F_{10,60}$=2.56, p<0.05; ChR2-eYFP: $F_{10,60}$=2.81, p<0.01; **D**, eYFP: chamber x experimental phase: $F_{10,50}$=4.81, p<0.001; ChR2-eYFP: $F_{10,70}$=6.14, p<0.001, ANOVA with Newman-Keuls post-hoc test). **E.** Cocaine CPP expression and reinstatement timeline with an additional saline test paired with photostimulation. When NAc photostimulation of VTA VGluT2- fibers was administered in the saline-paired chamber during a saline-priming test (ST), ChR2-eYFP mice (n=8) spent more time in the cocaine-paired chamber than eYFP control mice (bottom, n=8; eYFP: chamber x experimental phase: $F_{12,84}$=5.29, p<0.001; ChR2-eYFP: $F_{12,84}$=5.14, p<0.001, ANOVA with Newman-Keuls post-hoc test). * p<0.05, ** p<0.01, *** p<0.001, + p=0.07, against saline-paired chamber. Light-blue rectangles indicate photostimulation.

Supplementary figure 7. Selective targeting of VTA-VGluT2-TH, VTA-VGluT2-only, and VTA-TH-only neurons. A. Schematic of crossing between VGluT2::Cre and TH::Flp mice to generate double recombinase expressing VGluT2::Cre-TH::Flp mice and intra-VTA injection of INTRSECT AAV-C_{ON}/F_{ON}-ChR2-eYFP to target VGluT2-TH neurons, AAV-C_{ON}/F_{OFF}-ChR2-eYFP to target VGluT2-only neurons, and AAV-C_{OFF}/F_{ON}-ChR2-eYFP to target TH-only neurons. **B-C.** Low (**B**) and high (**C**) magnification images showing co-expression of VGluT2 mRNA and TH mRNA in eYFP-expressing neurons. **D-E.** Low (**D**) and high (**E**) magnification images showing expression of VGluT2 mRNA and lack of expression of TH mRNA in eYFP-expressing neurons. **F-G.** Low (**F**) and high (**G**) magnification images showing expression of TH mRNA and lack of expression of VGluT2 mRNA in eYFP-expressing neurons. **H.** Detection of VGluT2 and TH mRNAs within the subpopulation of VTA neurons co-expressing eYFP. **I.** Detection of VGluT2 mRNAs within the subpopulation of VTA neurons co-expressing eYFP. **J.** Detection of TH mRNAs within the subpopulation of VTA neurons co-expressing eYFP. The number of total counted neurons (“n”) is shown in each pie graph (3-4 mice/group).

Supplementary figure 8. VTA detection of INTRSECT viral injection sites and NAc location of optical probes. A. VTA injection of INTRSECT viral vectors and NAc shell optic fibers. **B.** Low magnification of VTA from mice injected with INTRSECT vectors (C_{ON}/F_{OFF}, top left; C_{ON}/F_{ON}, top right; C_{OFF}/F_{ON}, bottom) showing neurons expressing eYFP (green) and TH (red). **C.** NAc eYFP fibers (green) from VTA-VGluT2 (left), VTA-VGluT2-TH (middle), and VTA-TH (right) neurons and optic fiber placements. **D.** Maximal (blue) and minimal (red) rostro -caudal extension of INTRSECT viral injections within the VTA for control eYFP (left) and ChR2-eYFP (right) mice. **E.** NAc optic fiber placements in eYFP (left) and ChR2-eYFP (right) mice. Core, nucleus accumbens core; CPU, caudate putamen nucleus; cp, cerebral peduncle; IF, interfascicular nucleus; ml, medial lemniscus; mShell, nucleus accumbens medial shell; NAc, nucleus accumbens; PBP, parabrachial pigmented nucleus; PN, paranigral nucleus; RLi, rostro linear nucleus; VTA, ventral tegmental area; vtgx, ventral tegmental decussation.

Supplementary figure 9. Track plots for expression, and priming-induced reinstatement experiments using dual recombinase transgenic mice. A. VTA injection of INTRSECT viral vectors and NAc shell optic fibers. **B.** Cocaine CPP expression and reinstatement timeline. **C.** Track plots from an eYFP (top) and a ChR2-eYFP (bottom) mouse in which VTA-VGluT2-TH neurons were targeted, during pretest (PT), expression test (T), and cocaine test (CT) in the presence or absence of NAc photostimulation. Blue boxes indicate photostimulation. **D.** Track plots from an eYFP (top) and a ChR2-eYFP (bottom) mouse in which VTA-TH-only neurons were targeted, during pretest (PT), expression test (T), and cocaine test (CT) in the presence or absence of NAc photostimulation. Blue boxes indicate photostimulation. **E.** Traces from an individual neuron in response to NAc photostimulation of VTA-TH-VGluT2 fibers obtained before (control), and after sequential application of a D-AP5 (50 µM) and CNQX (10 µM) cocktail, followed by a SCH-23390 (10 µM) and sulpiride (10 µM) cocktail. Blue squares indicate light stimulation (5 ms). **F.** EPSC amplitude (pA) in control (aCSF: -18.33 ± 1.6 pA), + CNQX/D-AP5 (-3.23± 0.22 pA), + SCH-23390/sulpiride (-3.01 ± 0.33 pA) conditions (n=9 neurons from 6 mice). **G.** Traces from an individual neuron in response to NAc photostimulation of VTA-TH-only fibers obtained before (control) and after bath application of a SCH-23390 (10 µM) and sulpiride (10 µM) cocktail. Blue squares indicate light stimulation (5 ms). **H.** EPSC amplitude (pA) in control (aCSF: -19.54 ± 2.61 pA), + SCH-23390/sulpiride (-3.68 ± 0.34 pA) conditions (n=9 neurons from 6 mice). ***p<0.001 against control group.

Supplementary figure 10. NAc release of glutamate from VTA-VGluT2 fibers inhibits instrumental reinstatement of cocaine-seeking behavior. A. VTA injection of AAV5/2-DIO-eYFP or AAV5/2-DIO-ChR2-eYFP and NAc shell optic fibers. **B.** Cocaine self-administration and reinstatement timeline, showing training with cues and drug, extinction without cues or drug, and reinstatement conditions. Additional extinction sessions were run between each of the reinstatement conditions. **C.** Both ChR2-eYFP (n=14) and eYFP control mice (n=15) showed an increase in the number of total lever presses during the first day of extinction (day x lever: $F_{2,28}$=4.23, p<0.05, ANOVA with Newman-Keuls post-hoc test). ***

p<0.001, against the last day of extinction. **D.** Individual values for the reinstatement experiment showed in figure 4E. Light-blue rectangle indicates photostimulation.

Figure 11. Role of mesoaccumbal glutamatergic pathway in psychostimulant preference and seeking. NAc release of glutamate from VTA axon terminals that release glutamate without dopamine inhibits the expression, psychostimulant priming- and stress-induced reinstatement, but not the acquisition, of psychostimulant preference. In addition, it inhibits the priming-induced reinstatement of cocaine-seeking behavior.

Supplementary figures

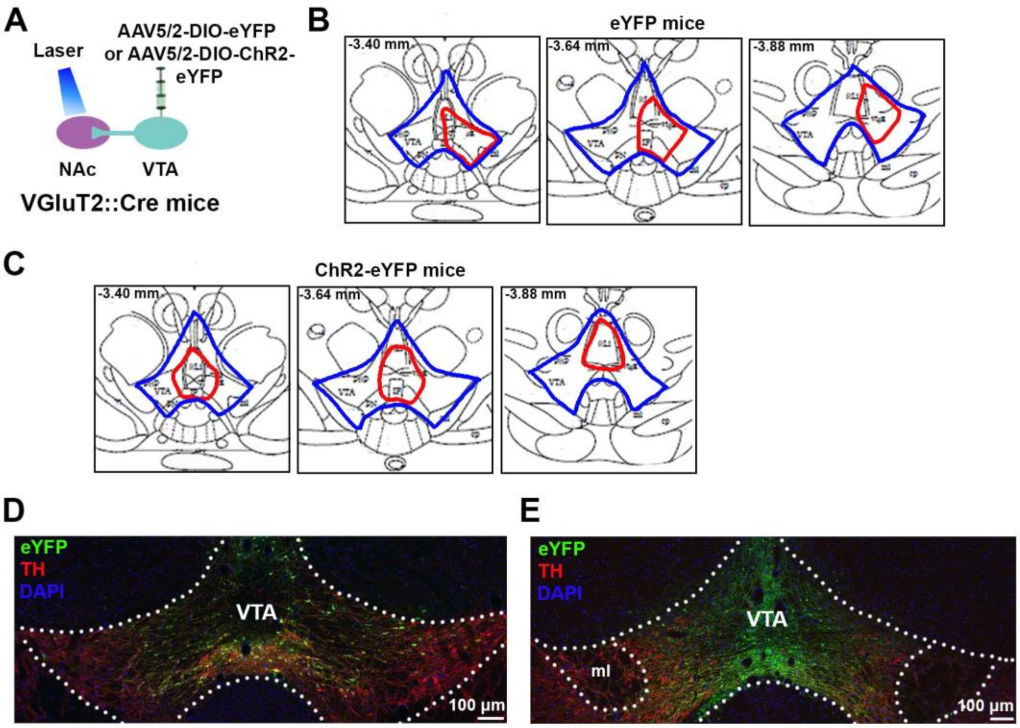

Supplementary figure 1. VTA detection of viral injection sites. A. VTA injection of AAV5/2-DIO-eYFP or AAV5/2-DIO-ChR2-eYFP and NAc shell optic fibers. **B-C.** Maximal (blue) and minimal (red) rostro-caudal extension of viral injections (**B**, eYFP; **C**, ChR2-eYFP) within the VTA. **D-E.** VTA immunofluorescence detection of VGluT2-expressing neurons (green) and TH (red) for an eYFP control mouse (**D**) and a ChR2-eYFP mouse (**E**). IF, interfascicular nucleus; ml, medial lemniscus; PBP, parabrachial pigmented nucleus; PN, paranigral nucleus; RLi, rostro linear nucleus; VTA, ventral tegmental area; vtgx, ventral tegmental decussation.

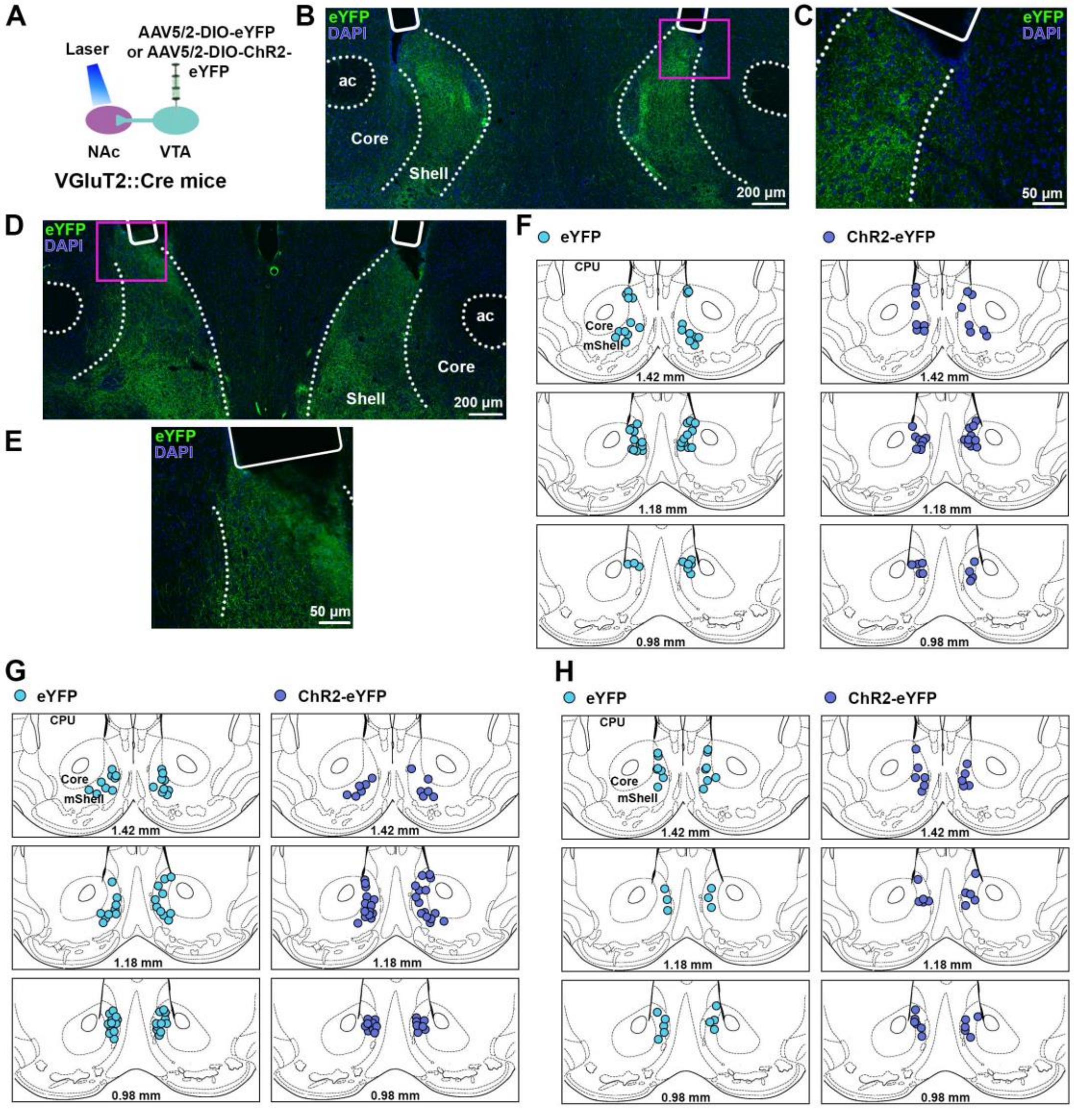

Supplementary figure 2. NAc location of optical probes. A. VTA injection of AAV5/2-DIO-eYFP or AAV5/2-DIO-ChR2-eYFP and NAc shell optic fibers. **B-E.** Low (**B**, **D**) and high (**C**, **E**) magnification NAc images showing eYFP fibers (green) from VTA-VGluT2 neurons and optic fiber placements for an eYFP control mouse (**B-C**) and a ChR2-eYFP mouse (**D-E**). **F.** NAc optic fiber placements in eYFP (left) and ChR2-eYFP (right) mice for figure 1 and supplementary figure 4. **G.** NAc optic fiber placements in eYFP (left) and ChR2-eYFP (right) mice for figures 2, 4, 5, supplementary figures 5 and 10. **H.** NAc optic fiber placements in eYFP (left) and ChR2-eYFP (right) mice for supplementary figure 6. Core, nucleus accumbens core; CPU, caudate putamen nucleus; cp, cerebral peduncle; mShell, nucleus accumbens medial shell.

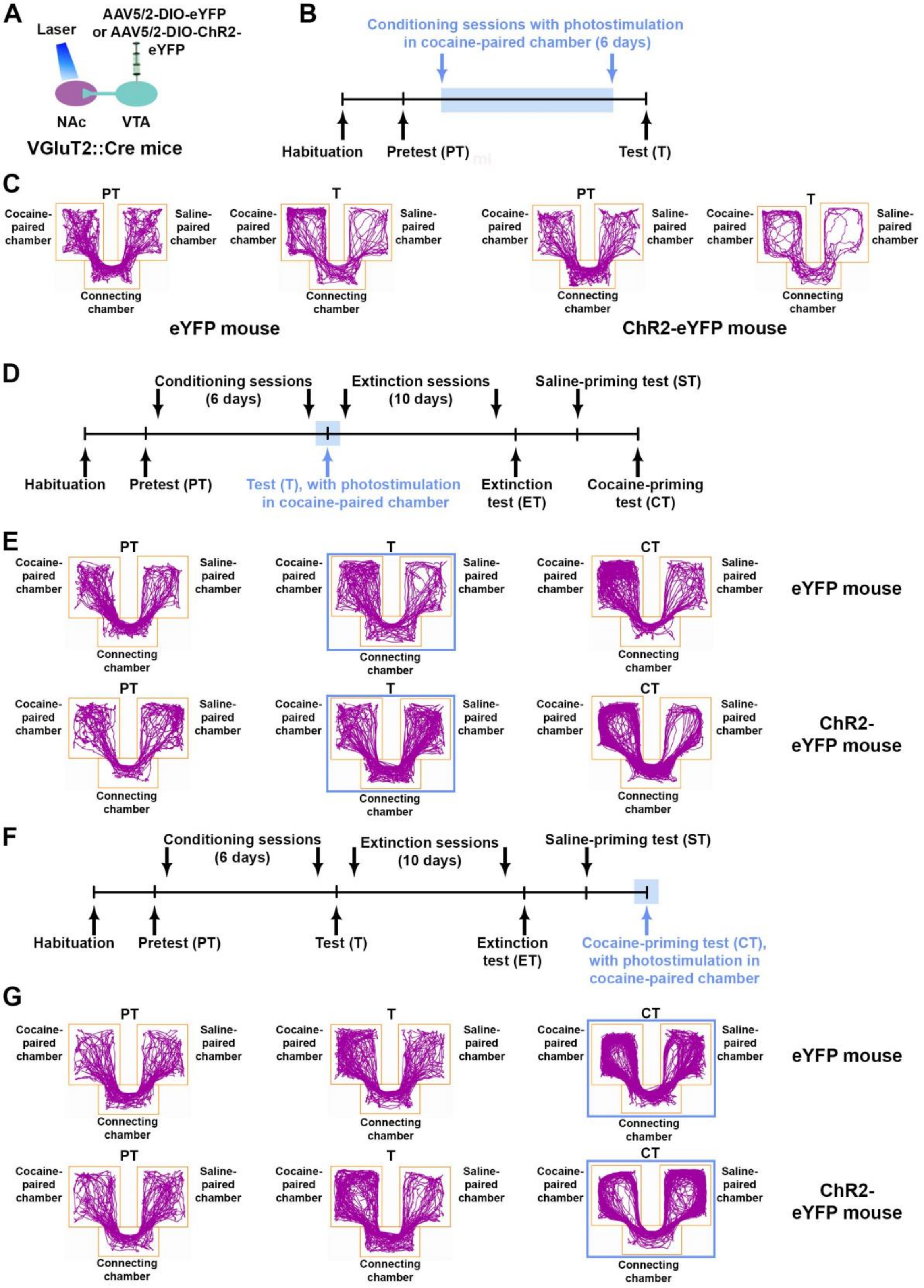

Supplementary figure 3. Track plots for acquisition, expression, and priming-induced reinstatement experiments. A. VTA injection of AAV5/2-DIO-eYFP or AAV5/2-DIO-ChR2-eYFP and NAc shell optic fibers. **B.** Cocaine CPP acquisition timeline. **C.** Track plots from an eYFP (left) and a ChR2-eYFP (right) mouse during pretest (PT) and expression test (T) after administration of NAc photostimulation during the conditioning sessions. **D.** Cocaine CPP expression timeline. **E.** Track plots from an eYFP (top) and a ChR2-eYFP (bottom) mouse during pretest (PT), expression test (T), and cocaine test (CT) in the presence or absence of NAc photostimulation. Blue boxes indicate photostimulation. **F.** Cocaine CPP reinstatement timeline. **G.** Track plots from an eYFP (top) and a ChR2-eYFP (bottom) mouse during pretest (PT), expression test (T), and cocaine test (CT) in the presence or absence of NAc photostimulation. Blue boxes indicate photostimulation.

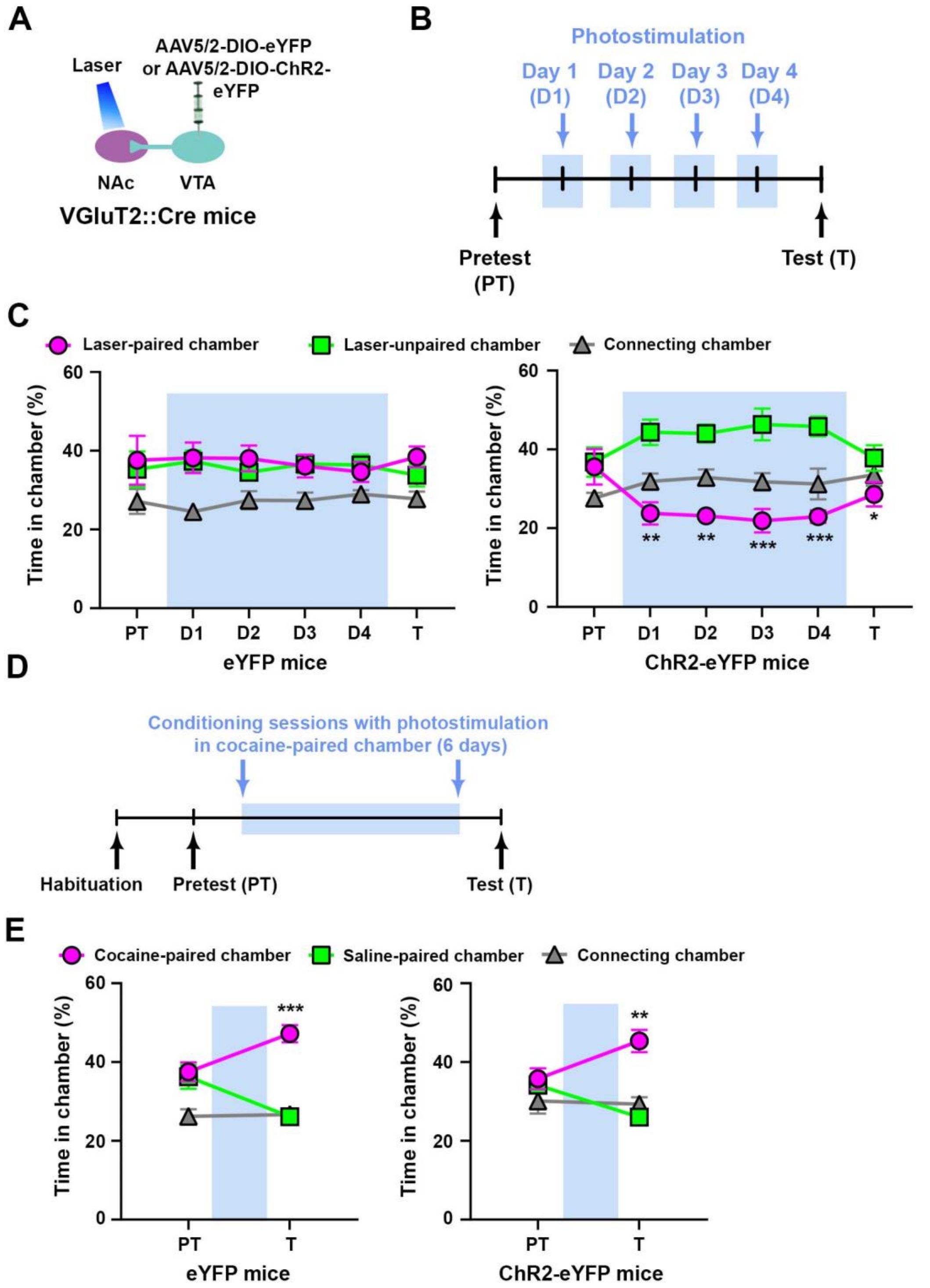

Supplementary figure 4. NAc release of glutamate from VTA-VGluT2 fibers induces aversion but does not modify the acquisition of cocaine CPP in the same cohort of mice. A. VTA injection of AAV5/2-DIO-eYFP or AAV5/2-DIO-ChR2-eYFP and NAc shell optic fibers. **B.** Laser-induced place aversion timeline. **C.** ChR2-eYFP mice (n = 6) spent significantly less time in the laser-paired chamber than eYFP control mice (n = 7) during and after NAc photostimulation sessions (eYFP: chamber x experimental phase: $F_{10,60}$=0.33, p=0.97, n.s.; ChR2-eYFP: chamber x experimental phase: $F_{10,50}$=2.98, p<0.01, ANOVA with Newman-Keuls post-hoc test). * p<0.05, ** p<0.01, *** p<0.001, against laser-unpaired chamber. **D.** Cocaine CPP acquisition timeline for the same mice from the laser-induced place aversion experiment. **E.** Both eYFP (n=7) and ChR2-eYFP mice (n=6) spent more time in the cocaine-paired chamber after the conditioning sessions (eYFP: chamber x experimental phase: $F_{2,12}$=7.56, p<0.01; ChR2-eYFP: $F_{2,10}$=6.26, p<0.05, ANOVA with Newman-Keuls post-hoc test). ** p<0.01, *** p<0.001, against saline-paired chamber. Light-blue rectangles indicate photostimulation.

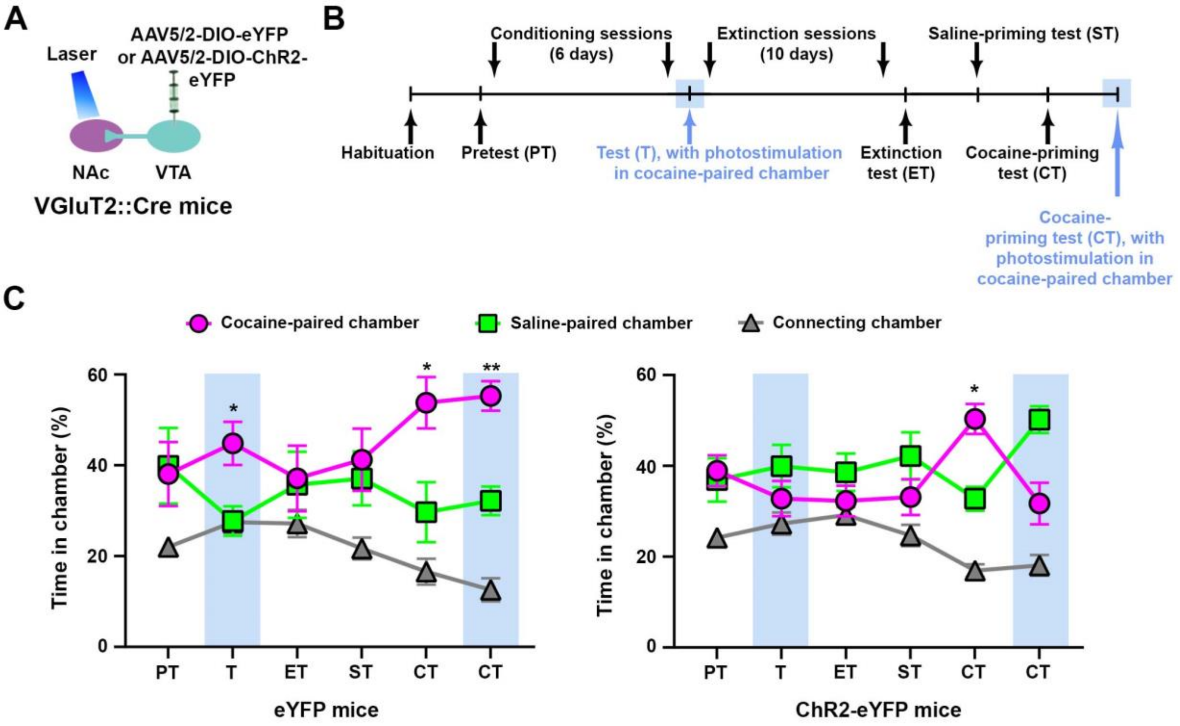

Supplementary figure 5. NAc shell release of glutamate from VTA-VGluT2 fibers inhibited the expression and reinstatement of cocaine CPP in female mice. A. VTA injection of AAV5/2-DIO-eYFP or AAV5/2-DIO-ChR2-eYFP and NAc shell optic fibers. **B.** Cocaine CPP expression and reinstatement timeline. **C.** ChR2-eYFP female mice (n=8) spent less time in the cocaine-paired chamber than eYFP female mice (n=6) during the expression test (T) paired with NAc photostimulation of VTA-VGluT2 fibers. While both groups showed reinstatement of cocaine CPP during the cocaine-priming test (CT) in the absence of photostimulation, only ChR2-eYFP female mice avoided the cocaine-paired chamber during the cocaine-priming test (CT) paired with photostimulation (eYFP: chamber x experimental phase: $F_{10,50}=2.86$, $p<0.01$; ChR2-eYFP: $F_{10,70}=4.40$, $p<0.001$, ANOVA with Newman-Keuls post-hoc test). * $p<0.05$, ** $p<0.01$, against saline-paired chamber. Light-blue rectangles indicate photostimulation.

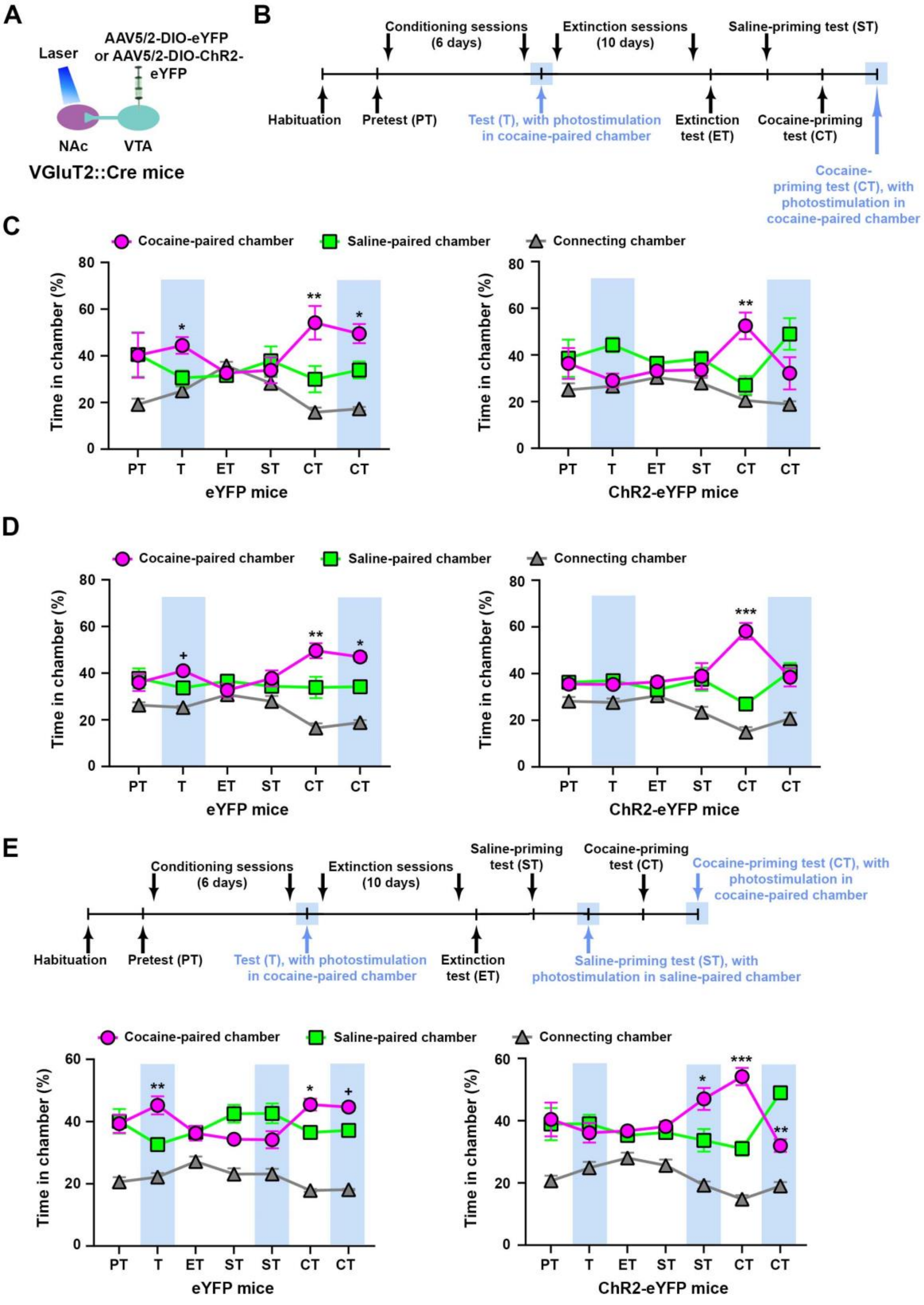

Supplementary figure 6. Suppression of cocaine-induced CPP expression and reinstatement by NAc release of glutamate from VTA-VGluT2 fibers does not depend on the dose of cocaine used. A. VTA injection of AAV5/2-DIO-eYFP or AAV5/2-DIO-ChR2-eYFP and NAc shell optic fibers. **B.** Cocaine CPP expression and reinstatement timeline. **C-D.** ChR2-eYFP mice injected with doses of 5 mg/kg (**C**, n=7) or 10 mg/kg (**D**, n=8) of cocaine spent less time in the cocaine-paired chamber than eYFP control mice (**C**, n=7; **D**, n=6) during the expression test (T) paired with NAc photostimulation of VTA-VGluT2 fibers. While both groups showed reinstatement of cocaine CPP during the cocaine-priming test (CT) in the absence of photostimulation, only ChR2-eYFP mice avoided the cocaine-paired chamber during the cocaine-priming test (CT) paired with photostimulation (**C**, eYFP: chamber x experimental phase: $F_{10,60}$=2.56, p<0.05; ChR2-eYFP: $F_{10,60}$=2.81, p<0.01; **D**, eYFP: chamber x experimental phase: $F_{10,50}$=4.81, p<0.001; ChR2-eYFP: $F_{10,70}$=6.14, p<0.001, ANOVA with Newman-Keuls post-hoc test). **E.** Cocaine CPP expression and reinstatement timeline with an additional saline test paired with photostimulation. When NAc photostimulation of VTA VGluT2- fibers was administered in the saline-paired chamber during a saline-priming test (ST), ChR2-eYFP mice (n=8) spent more time in the cocaine-paired chamber than eYFP control mice (bottom, n=8; eYFP: chamber x experimental phase: $F_{12,84}$=5.29, p<0.001; ChR2-eYFP: $F_{12,84}$=5.14, p<0.001, ANOVA with Newman-Keuls post-hoc test). * p<0.05, ** p<0.01, *** p<0.001, + p=0.07, against saline-paired chamber. Light-blue rectangles indicate photostimulation.

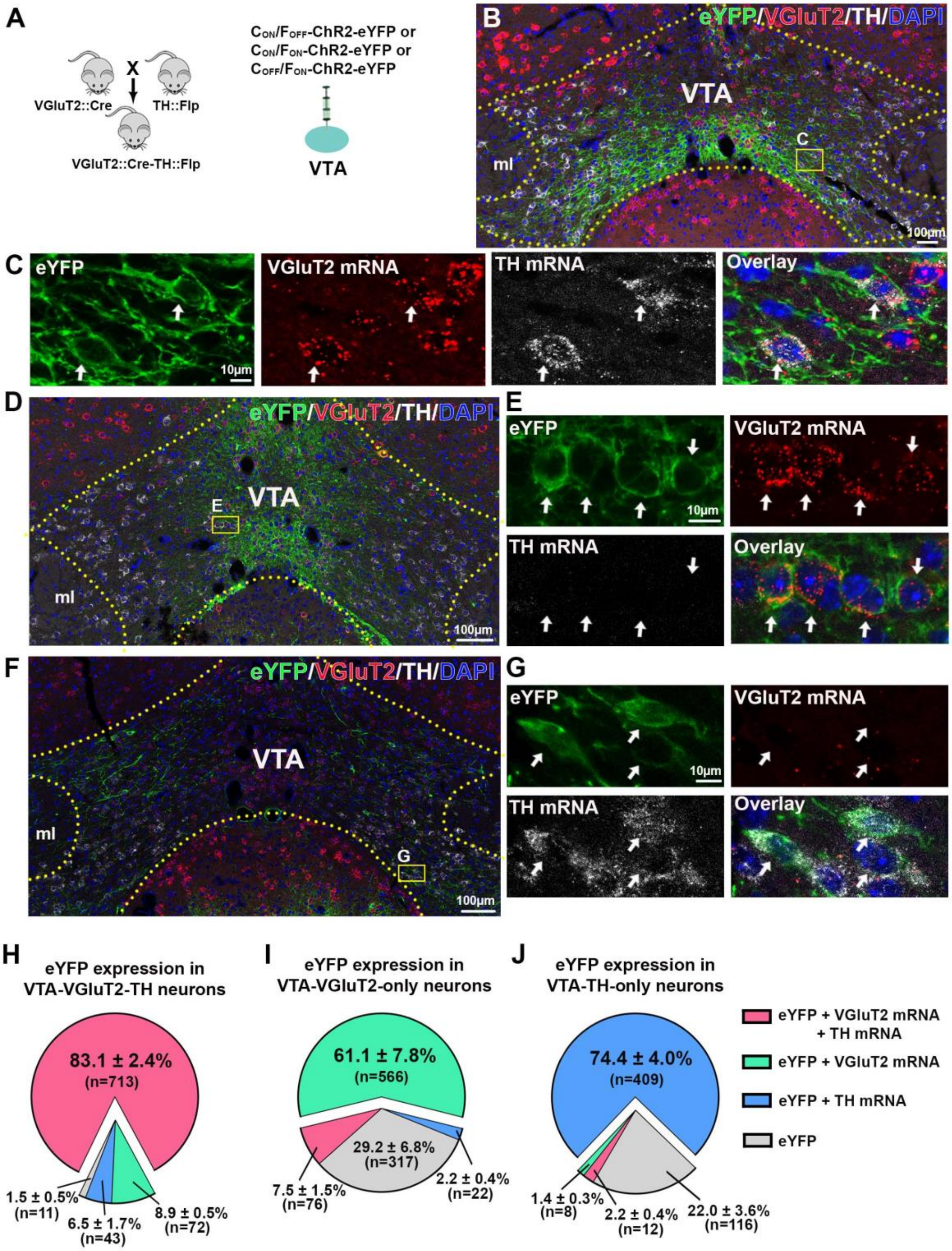

Supplementary figure 7. Selective targeting of VTA-VGluT2-TH, VTA-VGluT2-only, and VTA-TH-only neurons. A. Schematic of crossing between VGluT2::Cre and TH::Flp mice to generate double recombinase expressing VGluT2::Cre-TH::Flp mice and intra-VTA injection of INTRSECT AAV-C_{ON}/F_{ON}-ChR2-eYFP to target VGluT2-TH neurons, AAV-C_{ON}/F_{OFF}-ChR2-eYFP to target VGluT2-only neurons, and AAV-C_{OFF}/F_{ON}-ChR2-eYFP to target TH-only neurons. **B-C.** Low (**B**) and high (**C**) magnification images showing co-expression of VGluT2 mRNA and TH mRNA in eYFP-expressing neurons. **D-E.** Low (**D**) and high (**E**) magnification images showing expression of VGluT2 mRNA and lack of expression of TH mRNA in eYFP-expressing neurons. **F-G.** Low (**F**) and high (**G**) magnification images showing expression of TH mRNA and lack of expression of VGluT2 mRNA in eYFP-expressing neurons. **H.** Detection of VGluT2 and TH mRNAs within the subpopulation of VTA neurons co-expressing eYFP. **I.** Detection of VGluT2 mRNAs within the subpopulation of VTA neurons co-expressing eYFP. **J.** Detection of TH mRNAs within the subpopulation of VTA neurons co-expressing eYFP. The number of total counted neurons (“n”) is shown in each pie graph (3-4 mice/group).

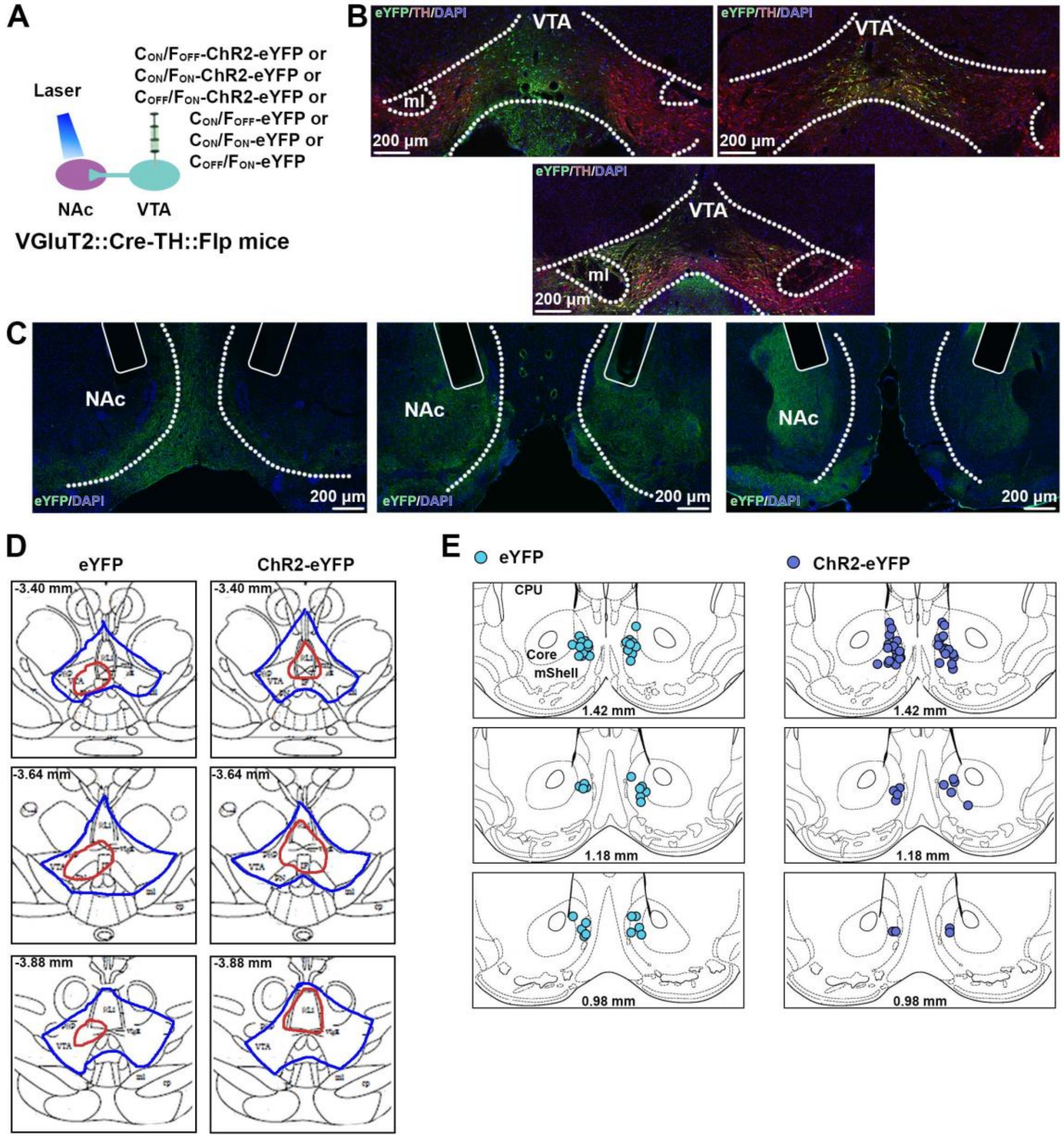

Supplementary figure 8. VTA detection of INTRSECT viral injection sites and NAc location of optical probes. A. VTA injection of INTRSECT viral vectors and NAc shell optic fibers. **B.** Low magnification of VTA from mice injected with INTRSECT vectors (C_{ON}/F_{OFF}, top left; C_{ON}/F_{ON}, top right; C_{OFF}/F_{ON}, bottom) showing neurons expressing eYFP (green) and TH (red). **C.** NAc eYFP fibers (green) from VTA-VGluT2 (left), VTA-VGluT2-TH (middle), and VTA-TH (right) neurons and optic fiber placements. **D.** Maximal (blue) and minimal (red) rostro -caudal extension of INTRSECT viral injections within the VTA for control eYFP (left) and ChR2-eYFP (right) mice. **E.** NAc optic fiber placements in eYFP (left) and ChR2-eYFP (right) mice. Core, nucleus accumbens core; CPU, caudate putamen nucleus; cp, cerebral peduncle; IF, interfascicular nucleus; ml, medial lemniscus; mShell, nucleus accumbens medial shell; NAc, nucleus accumbens; PBP, parabrachial pigmented nucleus; PN, paranigral nucleus; RLi, rostro linear nucleus; VTA, ventral tegmental area; vtgx, ventral tegmental decussation.

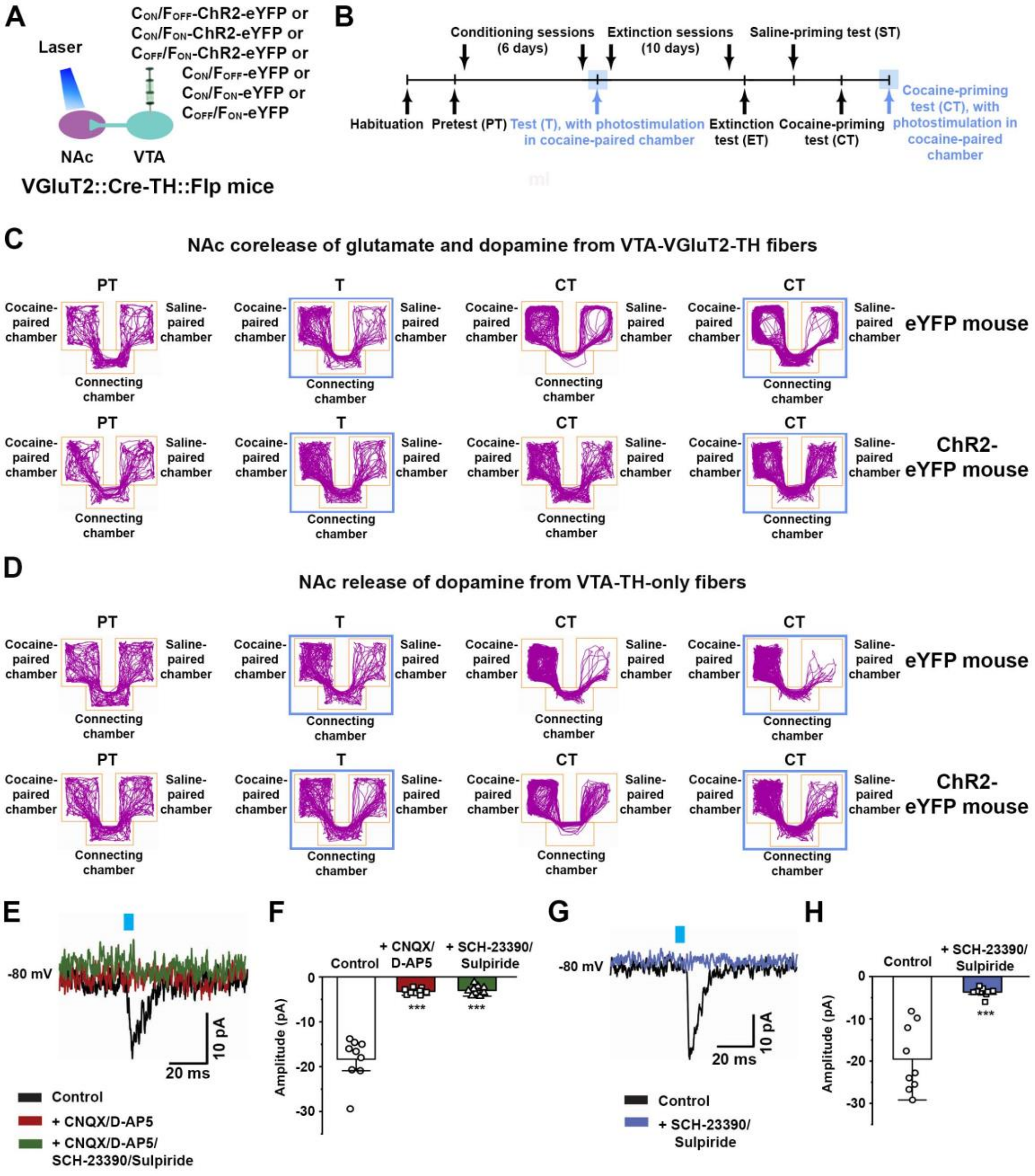

Supplementary figure 9. Track plots for expression, and priming-induced reinstatement experiments using dual recombinase transgenic mice. A. VTA injection of INTRSECT viral vectors and NAc shell optic fibers. **B.** Cocaine CPP expression and reinstatement timeline. **C.** Track plots from an eYFP (top) and a ChR2-eYFP (bottom) mouse in which VTA-VGluT2-TH neurons were targeted, during pretest (PT), expression test (T), and cocaine test (CT) in the presence or absence of NAc photostimulation. Blue boxes indicate photostimulation. **D.** Track plots from an eYFP (top) and a ChR2-eYFP (bottom) mouse in which VTA-TH-only neurons were targeted, during pretest (PT), expression test (T), and cocaine test (CT) in the presence or absence of NAc photostimulation. Blue boxes indicate photostimulation. **E.** Traces from an individual neuron in response to NAc photostimulation of VTA-TH-VGluT2 fibers obtained before (control), and after sequential application of a D-AP5 (50 µM) and CNQX (10 µM) cocktail, followed by a SCH-23390 (10 µM) and sulpiride (10 µM) cocktail. Blue squares indicate light stimulation (5 ms). **F.** EPSC amplitude (pA) in control (aCSF: -18.33 ± 1.6 pA), + CNQX/D-AP5 (-3.23± 0.22 pA), + SCH-23390/sulpiride (-3.01 ± 0.33 pA) conditions (n=9 neurons from 6 mice). **G.** Traces from an individual neuron in response to NAc photostimulation of VTA-TH-only fibers obtained before (control) and after bath application of a SCH-23390 (10 µM) and sulpiride (10 µM) cocktail. Blue squares indicate light stimulation (5 ms). **H.** EPSC amplitude (pA) in control (aCSF: -19.54 ± 2.61 pA), + SCH-23390/sulpiride (-3.68 ± 0.34 pA) conditions (n=9 neurons from 6 mice). ***p<0.001 against control group.

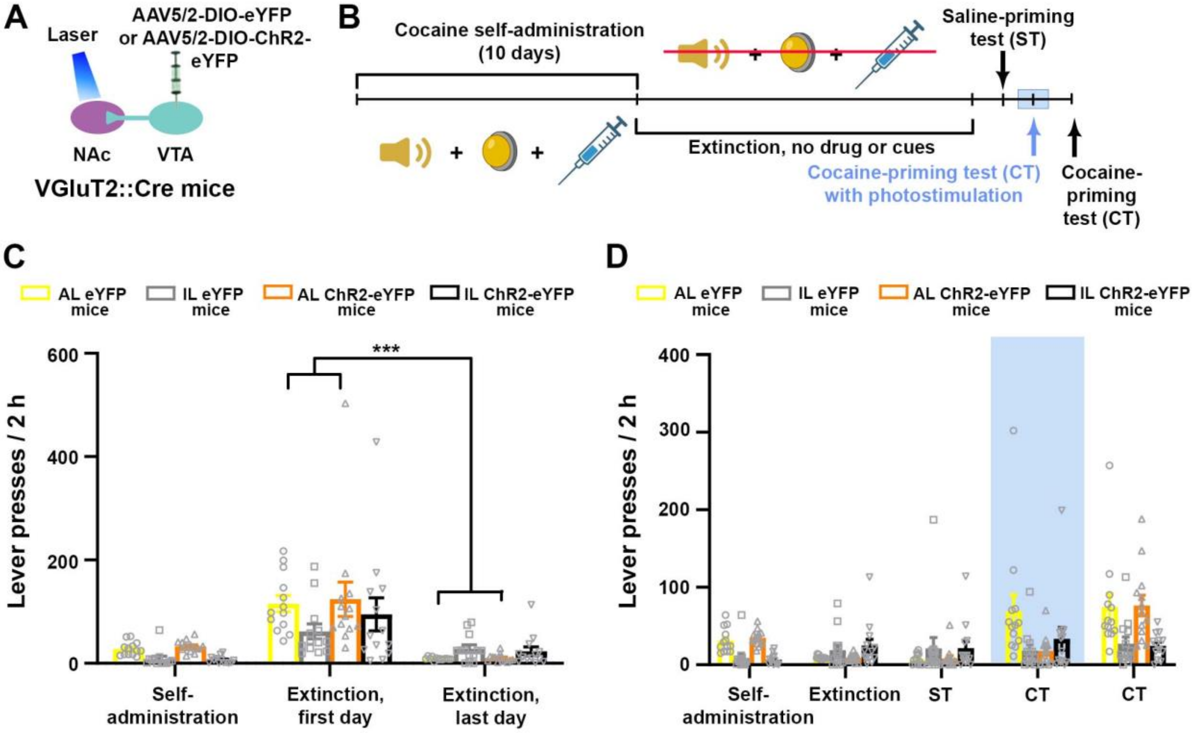

Supplementary figure 10. NAc release of glutamate from VTA-VGluT2 fibers inhibits instrumental reinstatement of cocaine-seeking behavior. A. VTA injection of AAV5/2-DIO-eYFP or AAV5/2-DIO-ChR2-eYFP and NAc shell optic fibers. **B.** Cocaine self-administration and reinstatement timeline, showing training with cues and drug, extinction without cues or drug, and reinstatement conditions. Additional extinction sessions were run between each of the reinstatement conditions. **C.** Both ChR2-eYFP (n=13) and eYFP control mice (n=13) showed an increase in the number of total lever presses during the first day of extinction (day x lever: $F_{2,48}=6.99$, $p<0.01$, ANOVA with Newman-Keuls post-hoc test). *** $p<0.001$, against the last day of extinction. **D.** Individual values for the reinstatement experiment showed in figure 4E. Light-blue rectangle indicates photostimulation.

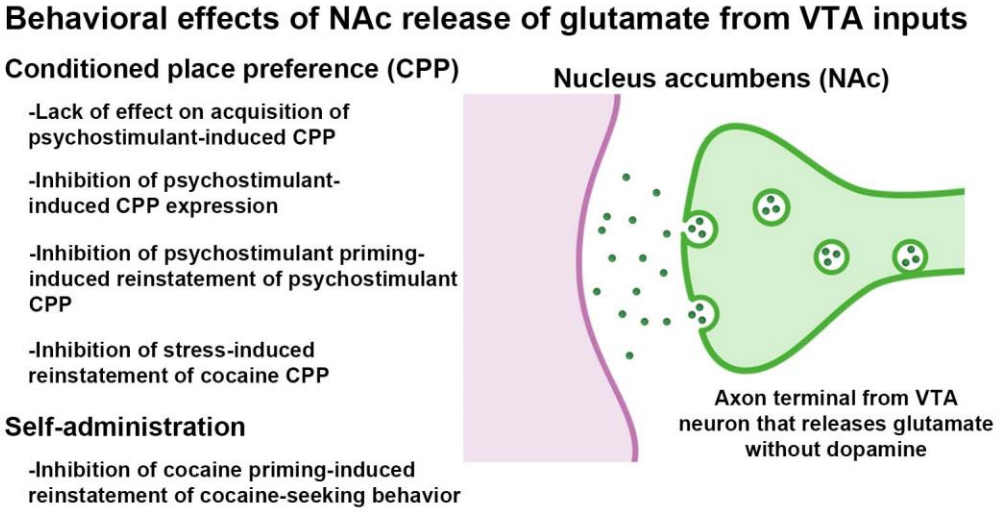

Figure 11. Role of mesoaccumbal glutamatergic pathway in psychostimulant preference and seeking. NAc release of glutamate from VTA axon terminals that release glutamate without dopamine inhibits the expression, psychostimulant priming- and stress-induced reinstatement, but not the acquisition, of psychostimulant preference. In addition, it inhibits the priming-induced reinstatement of cocaine-seeking behavior.
